# Hydrogen Peroxide Scavenging Restores N-Type Calcium Channels in Cardiac Vagal Postganglionic Neurons and Mitigates Myocardial Infarction-Evoked Ventricular Arrhythmias in Type 2 Diabetes Mellitus

**DOI:** 10.3389/fcvm.2022.871852

**Published:** 2022-04-25

**Authors:** Dongze Zhang, Huiyin Tu, Wenfeng Hu, Bin Duan, Matthew C. Zimmerman, Yu-Long Li

**Affiliations:** ^1^Department of Emergency Medicine, University of Nebraska Medical Center, Omaha, NE, United States; ^2^Mary and Dick Holland Regenerative Medicine Program, Division of Cardiology, Department of Internal Medicine, University of Nebraska Medical Center, Omaha, NE, United States; ^3^Department of Cellular and Integrative Physiology, University of Nebraska Medical Center, Omaha, NE, United States

**Keywords:** calcium channel, cardiac vagal neuron, hydrogen peroxide, myocardial infarction, type 2 diabetes, ventricular arrhythmia

## Abstract

**Objective:**

Withdrawal of cardiac vagal activity is associated with ventricular arrhythmia-related high mortality in patients with type 2 diabetes mellitus (T2DM). Our recent study found that reduced cell excitability of cardiac vagal postganglionic (CVP) neurons is involved in cardiac vagal dysfunction and further exacerbates myocardial infarction (MI)-evoked ventricular arrhythmias and mortality in T2DM. However, the mechanisms responsible for T2DM-impaired cell excitability of CVP neurons remain unclear. This study tested if and how elevation of hydrogen peroxide (H_2_O_2_) inactivates CVP neurons and contributes to cardiac vagal dysfunction and ventricular arrhythmogenesis in T2DM.

**Methods and Results:**

Rat T2DM was induced by a high-fat diet plus streptozotocin injection. Local *in vivo* transfection of adenoviral catalase gene (Ad.CAT) successfully induced overexpression of catalase and subsequently reduced cytosolic H_2_O_2_ levels in CVP neurons in T2DM rats. Ad.CAT restored protein expression and ion currents of N-type Ca^2+^ channels and increased cell excitability of CVP neurons in T2DM. Ad.CAT normalized T2DM-impaired cardiac vagal activation, vagal control of ventricular function, and heterogeneity of ventricular electrical activity. Additionally, Ad.CAT not only reduced the susceptibility to ventricular arrhythmias, but also suppressed MI-evoked lethal ventricular arrhythmias such as VT/VF in T2DM.

**Conclusions:**

We concluded that endogenous H_2_O_2_ elevation inhibited protein expression and activation of N-type Ca^2+^ channels and reduced cell excitability of CVP neurons, which further contributed to the withdrawal of cardiac vagal activity and ventricular arrhythmogenesis in T2DM. Our current study suggests that the H_2_O_2_-N-type Ca^2+^ channel signaling axis might be an effective therapeutic target to suppress ventricular arrhythmias in T2DM patients with MI.

## Introduction

Diabetes is a major public health problem worldwide and has become a leading cause of mortality (1–3), which is expected to affect more than 700 million adults by 2045 (4), with most having type 2 diabetes mellitus (T2DM, 90–95% of diabetic population) (5). Myocardial infarction (MI)-related ventricular arrhythmia is the primary cause of mortality in T2DM patients (3, 6). Although well-known therapies, including intensive glycemic control over time, have been noted in T2DM patients, these treatments fail to reduce MI-related mortality in T2DM patients (7, 8), and excess risk of death still exists (3, 9, 10). Withdrawal of cardiac vagal (parasympathetic) activity is a common complication (9, 11, 12) and is associated with arrhythmia-related sudden cardiac death in T2DM patients (13–16).

Regulation of cardiac vagal activity can be integrated by the regulatory circuitry at multiple levels, including vagal nerve afferent at baroreceptors, central components, and efferent components (cardiac vagal ganglia) (17). Although structural and functional alterations in every site of the circuitry could cause attenuation of cardiac vagal activity, impairment of cardiac vagal ganglia might be an important factor for the withdrawal of cardiac vagal activity in T2DM, because: (1) cardiac vagal ganglionic neurons provide local neural coordination independent of higher brain centers (18); (2) acetylcholine (ACh) release from cardiac vagal neurons is blunted in T2DM patients (19); (3) our previous study found that cell excitability of cardiac vagal postganglionic (CVP) neurons was reduced due to lower expression of voltage-gated Ca^2+^ channels in T2DM rats (20). Cardiac vagal ganglia are divided into the sinoatrial ganglion and atrioventricular ganglion (AVG) (21), and the ventricle only receives projection of vagal nerve terminals from the AVG (22). Our previous study found that reduced N-type Ca^2+^ channels (Ca_v_2.2) and cell excitability of CVP neurons contribute to the withdrawal of ventricular vagal function in T2DM (23). More importantly, T2DM-reduced cell excitability of CVP neurons exacerbated MI-evoked ventricular arrhythmias and high mortality rate in T2DM (24). However, the mechanisms responsible for low-expression and inactivation of N-type Ca^2+^ channels in CVP neurons are unclear.

In physiological conditions, reactive oxygen species (ROS) such as hydrogen peroxide [H_2_O_2_, the most stable of ROS (25, 26)] play an essential role in cell proliferation and differentiation, signaling transduction, gene expression, etc. (27–29). However, excessive ROS production could destroy cellular complexes (30), leading to the pathogenesis of T2DM (31, 32). Indeed, growing evidence demonstrates that diabetes induces overproduction of ROS, including H_2_O_2_, in multiple tissues and cells through various signaling pathways (33–35). Considering that some previous studies demonstrated that H_2_O_2_ could acutely modulate voltage-gated Ca^2+^ channels (36–38), in the present study, we used *in vivo* transfection of adenoviral catalase gene (Ad.CAT) in the AVG to reveal the involvement of H_2_O_2_ in CVP neuronal dysfunction, ventricular vagal abnormality, and MI-evoked ventricular arrhythmias in T2DM.

## Materials and Methods

The study conformed to guidelines for the Care and Use of Laboratory Animals and was approved by the Institutional Animal Care and Use Committee (IACUC, NO.18-023-04-FC) at the University of Nebraska Medical Center. As an analgesic, buprenorphine (0.05 mg/kg, s.c., Reckitt Benckiser Pharmaceuticals Inc., Richmond, VA, United States) was given for three post-operative days in all survival surgical procedures. After *in vivo* experiments were completed, rats were euthanized with 0.39 ml/kg of Fatal-Plus euthanasia solution (about 150 mg/kg pentobarbital, i.p., Vortech Pharmaceuticals, Dearborn, MI, United States).

### Experimental Design, Timeline, and Interventions

In the current study, 147 male Sprague-Dawley rats (6–7 weeks of age, weighting 180–200 g) were randomly assigned to one of two groups, including sham and T2DM. T2DM was induced by a combination of high-fat diet with low-dose streptozotocin (STZ) injection, as fully described below. All experiments were performed at 12–14 weeks of feeding with either standard chow diet (sham rats) or high-fat diet (T2DM rats). Implantation of ECG radiotelemetry was performed at the 12th week. T2DM rats were then divided into three subgroups for different interventions, including T2DM, T2DM+adenoviral vector (Ad.Empty), and T2DM+Ad.CAT. Saline, Ad.Empty, or Ad.CAT was microinjected into the AVG at the 12th week. Terminal experiments, including measurements for inducibility of ventricular arrhythmia and vagal control of ventricular function, were performed in anesthetized rats at 1week after gene transfection (Supplementary Figure 1). In addition, to access the cardiac autonomic function and ventricular electrical activities in the conscious state, heart rate variability (HRV) and ventricular arrhythmogenesis-related ECG markers were analyzed from 24-h radiotelemetry ECG recording in conscious rats at 1 week after gene transfection. After *in vivo* experiments were completed, rats were euthanized, and then AVGs were isolated to perform the *in vitro* experiments, including measurement of catalase activity, reverse–phase protein microarray, intracellular H_2_O_2_ and Ca^2+^ images, and whole-cell patch-clamp recording for Ca^2+^ currents and action potentials (APs). Moreover, MI-induced ventricular arrhythmias were also evaluated in all groups of conscious rats, in which MI was achieved by the ligation of the left anterior descending coronary artery (LAD). Continuous 24-h ECG recording was started immediately after LAD ligation. Incidence and duration of ventricular tachycardia/fibrillation (VT/VF) were quantified within 24-h after MI in all groups of conscious rats (Supplementary Figure 1).

### T2DM Rat Model

All rats were housed two per cage under controlled temperature, humidity, and a 12:12-h dark-light cycle. They were provided water and rat chow *ad libitum*. T2DM was induced by a combination of high-fat diet with streptozotocin (STZ) treatment, as previously described (20, 39). The rats were fed a high-fat diet consisting of 42% fat, 42.7% carbohydrate, and 15.2% protein (Harlan Teklad adjusted fat diet, Harlan Teklad, Madison, WI) for 4 weeks. The rats were then injected with STZ (30 mg/kg, i.p.) and continued with the high-fat diet. In the sham group, the rats were fed a standard chow diet consisting of 13% fat, 53% carbohydrate, and 34% protein (Harlan Teklad sterilizable rodent diet; Harlan Teklad, Madison, WI). All experiments were performed at 12–14 weeks of feeding with either standard chow diet or high-fat diet because our previous study revealed the characteristics of T2DM (hyperlipidemia, insulin resistance, and hyperglycemia) during this period (20). Nineteen rats were excluded from the study, in which 4 rats died during the progression of T2DM, and 15 rats were not considered as T2DM due to insufficient fasting blood glucose (<250 mg/dl). Basal metabolic characteristics from sham and T2DM rats were summarized in Supplementary Table 1.

### *In vivo* Gene Transfection of Ad.CAT or Ad.Empty Into the AVG

Rats were anesthetized with 2% isoflurane (Butler Schein Animal Health, Dublin, OH, USA), artificially ventilated, and then kept in right lateral recumbent position. A left posterolateral thoracotomy was performed through the 3rd left intercostal space. After the AVG located at the junction of the inferior pulmonary veins and left atrium was identified, 2 μl of saline, Ad.CAT [1 × 10^10^ pfu/ml, University of Iowa, Iowa City, IA (40)], or adenoviral vector control (Ad.Empty, 1 × 10^10^ pfu/ml, University of Iowa, Iowa City, IA) was microinjected into the AVG by a glass micropipette connected to a WPI Nanoliter 2000 microinjector. After microinjection, the chest was closed, and the experiments were performed at least 1 week after gene transfection to guarantee the overexpression of catalase. Ad.CAT purchased from the University of Iowa did not have a fluorescent tag. However, based on our previous study that microinjection of viral-GFP into the AVG induced GFP expression in almost all CVP neurons (23), we confirm the efficacy of virus infection and proper microinjection into the AVG. In addition, to confirm the specificity of adenoviral gene transfection, Ad. EGFP (adenovirus-enhanced green fluorescent protein) was microinjected into the AVG. At 1 week after gene transfection, expression of EGFP is restricted only in the AVG area but not in the left atrial myocardium (Supplementary Figure 2).

### Implantation of the ECG Radiotelemetry and ECG Recoding in Conscious Rats

Implantation of the ECG telemeter (Millar Instruments, Houston, TX, USA) was performed as described previously (41–43). After laparotomy was performed at the Linea Alba under anesthetized condition (2% isoflurane), an ECG transmitter was placed into the abdominal cavity and secured to the abdominal wall at the best position for signal communication and battery recharging, and the bipolar electrodes were then tunneled subcutaneously. In accordance with the Millar User Manual for ECG recording, the positive electrode was attached to the underlying tissue near the left side of the xiphoid process and the negative one was secured in the upper sternal midline. The electrodes were kept together and run alongside one another as far as practical for a significant reduction in the electrical noise during the recording. All incisions were sutured in two layers.

At 1–2 weeks after implantation of the ECG radiotelemetry, the rat was placed on a SmartPad receiver (Millar Instruments, Houston, TX, USA) for a 24-h continuous ECG recording in the conscious condition. Real-time ECG signals were digitalized and analyzed by PowerLab 8/30 Data Acquisition System with LabChart 8 software and ECG analysis module (AD Instruments, Colorado Springs, CO, USA).

### Measurements of Ventricular Electrical Activity and the HRV in Conscious Rats

To quantify the ventricular electrical activity, ventricular arrhythmogenesis-related ECG markers, including QT and corrected QT (QTc) intervals, QT and QTc dispersions, as well as T-peak to T-end (Tpe) interval, were calculated from ECG segments during the 24-h recording in conscious rats, as described previously (23, 44). QTc interval was calculated by Bazett's formula (QT/$\sqrt{RR}$, where RR is RR interval) (45). As an index of the spatial dispersion of the ventricular repolarization, QT and QTc dispersions were calculated by equations: QT dispersion = QT_max_-QT_min_ and QTc dispersion = QTc_max_-QTc_min_, where QT_max_ and QTc_max_ are the maximum QT interval and the maximum QTc interval; QT_min_ and QTc_min_ are the minimum QT interval and the minimum QTc interval. T-peak to T-end (Tpe) interval, another marker of transmural dispersion of the ventricular repolarization, was calculated and served as an ECG marker of ventricular arrhythmias (46–48).

Because the HRV is a commonly used index for determination of the autonomic function in T2DM patients in the clinic (49, 50), it was employed to evaluate the autonomic function in conscious rats in the current study. The HRV, including low-frequency power (LF) from 0.2 to 0.75 Hz, and high-frequency power (HF) from 0.75 to 2.5 Hz, was analyzed and averaged from eight ECG segments during the 24-h ECG recording in conscious rats (51–53).

### Measurement of Susceptibility to Ventricular Tachyarrhythmia in Anesthetized Rats

The rat was anesthetized with a mixture of 800 mg/kg urethane and 40 mg/kg α-chloralose and artificially ventilated. Surface lead-II ECG was recorded using subcutaneous electrodes connected to a biological amplifier (AD Instruments, Colorado Springs, CO, USA). A left thoracotomy was performed in the 4th intercostal space to expose the heart. After the pericardium was carefully removed, a bipolar platinum stimulating electrode was placed on the right ventricular outflow tract for electrical stimulation (54). Programmed electrical stimulation (PES) was performed by a programmed electrical stimulator (Digital Pulse Generator 1831; WPI, USA) and an isolator (A320R Isostim Stimulator; WPI, USA). The pulse current output was set to twice the capture threshold with a 2-ms pulse width. A train of eight stimuli (8 × S1) at a 120 ms cycle length followed by an extra-stimulus (S2) was applied to determine the ventricular effective refractory period. The S1–S2 interval was gradually reduced in steps of 2 ms (starting from 90 ms) until the ventricular effective refractory period was identified (55). Based on the ventricular effective refractory period, a programmed stimulation protocol combined by single (S2), double (S3), or triple extra- stimulus (S4) after a train of eight stimuli (8 × S1) was designed to induce ventricular tachyarrhythmia as described previously (43, 54, 56). The end point of ventricular pacing was the induction of ventricular tachyarrhythmia. Ventricular tachyarrhythmia was considered non-inducible when either PES failed to induce premature ventricular beats or self-terminated ventricular premature beats <6. Ventricular tachyarrhythmia was deemed to be non-sustained when it lasted ≤ 15 beats and sustained when it lasted > 15 beats before spontaneously terminating (57, 58). The inducibility of ventricular tachyarrhythmia was quantified by a quotient of ventricular arrhythmia score as described previously (54, 57). Zero, non-inducible preparations; 1, non-sustained tachyarrhythmias induced with 3 extra-stimuli; 2, sustained tachyarrhythmias induced with 3 extra-stimuli; 3, non-sustained tachyarrhythmias induced with 2 extra-stimuli; 4, sustained tachyarrhythmias induced with 2 extra-stimuli; 5, non-sustained tachyarrhythmias induced with 1 extra-stimulus; 6, sustained tachyarrhythmias induced with 1 extra-stimulus; 7, tachyarrhythmias induced during a train of 8 stimuli (8 × S1) at a basic cycle length of 120 ms; 8, the heart stopped before PES.

### Measurements of Acute MI-Induced Ventricular Arrhythmias in Conscious Rats

Given that acute MI-related ventricular arrhythmia is the most common cause of mortality in T2DM patients (3, 6), MI achieved by ligation of the LAD was used to induce the ventricular arrhythmia in the current study. Briefly, under the anesthetized condition (2% isoflurane) and mechanical ventilation, the left thoracotomy was performed at the 4th intercostal space to expose the heart. After pericardium was removed, the LAD was ligated with a 6-0 silk suture, just below its exit from the aorta, between the pulmonary artery outflow tract and left atrium. Then, the chest and surgical incision were closed. To quantification of ventricular arrhythmic events, continuous 24-h ECG recording was immediately started once the animal woke up from the surgery of LAD ligation. Incidence and duration of VT/VF were quantified within 24-h after MI in conscious rats. The cumulative duration of VT/VF was manually counted during 24-h continuous ECG recording. VT was defined as premature ventricular contractions lasting ≥4 beats. VF was defined as rapid, irregular QRS complexes.

### Measurement of Hemodynamics and Vagal Control of Ventricular Function

Under the anesthetized condition (a mixture of 800 mg/kg urethane and 40 mg/kg α-chloralose, i.p.) and mechanical ventilation, the left femoral artery was cannulated with a polyethylene-50 catheter to monitor blood pressure and heart rate. A Millar pressure transducer (SPR 524; size, 3.5-Fr; Millar Instruments, Houston, TX, USA) was slowly inserted into the right carotid artery and carefully advanced to the left ventricle to measure left ventricular systolic pressure (LVSP) and the maximum rate of increase of left ventricular pressure (LV dP/dt_max_). Hemodynamic data were recorded by PowerLab 8/30 Data Acquisition System with LabChart 8 software (AD Instruments, Colorado Springs, CO, USA) and summarized in Supplementary Table 3. To determine the vagal control of ventricular function, bilateral cervical vagal nerves, cervical sympathetic nerves, and aortic depressor nerves were isolated and transected to avoid the influence of the arterial baroreflex. Then, the peripheral end of the left vagal nerve was placed on a bipolar stimulating electrode for vagal efferent nerve stimulation (VNS), which was achieved by a Grass S9 stimulator (Grass Instruments, Quincy, MA, USA) with 10 s of constant-frequency stimulation (0.1 ms pulse duration and intensity of 7.5 V at 1–100 Hz). Changes of LVSP and LV dP/dt_max_ in response to different frequencies of VNS were severed as the index of vagal control of ventricular function and were recorded by PowerLab 8/30 data acquisition system with LabChart 8 software.

### Isolation of CVP Neurons and Whole-Cell Patch-Clamp Recording for Ca^2+^ Currents and APs

After *in vivo* experiments were performed, AVG was exposed and removed quickly. CVP neurons were isolated by a two-step enzymatic digestion protocol as described previously (20, 23, 59, 60). Briefly, isolated AVGs were placed in ice-cold modified Tyrode's solution (mM): 140 NaCl, 5 KCl, 10 HEPES, 5 glucose. The AVG was then minced into small pieces with microscissors and incubated with a modified Tyrode's solution containing 0.1% collagenase (type IV, C5138, Sigma-Aldrich) and 0.1% trypsin (type II, T7409, Sigma-Aldrich) for 30 min at 37°C. The tissue was then transferred to a modified Tyrode's solution containing 0.2% collagenase and 0.5% bovine serum albumin for 30 min of incubation at 37°C. The isolated CVP neurons were cultured in culture medium at 37°C in a humidified atmosphere of 95% air-5% CO_2_ for 4–8-h before patch-clamp experiments. The culture medium consisted of a 50/50 mixture of Delbecco's modified Eagle's medium (DMEM) and Ham's F12 medium supplemented with antibiotics and 10% fetal serum.

Voltage-gated Ca^2+^ currents and APs were recorded in CVP neurons by the whole-cell patch-clamp technique using Axopatch 200B patch-clamp amplifier (Axon Instruments) (20, 23, 24). Resistance of the patch pipette was 4–6 MΩ when filled with following solution (in mM): 120 CsCl, 1 CaCl2, 40 HEPES, 11 EGTA, 4 MgATP, 0.3 Tris-GTP, 14 creatine phosphate, and 0.1 leupeptin (pH 7.3; 305 mOsm/L). The extracellular solution consisted of (in mM): 140 TEA-Cl, 5 BaCl2, 1 MgCl2, 10 HEPES, 0.001 TTX, 2 4-AP, and 10 glucose (pH 7.4; 310 mOsm/L). Series resistance of 5–13 MΩ was electronically compensated 30–80%. Junction potential was calculated to be +7.9 mV using pCLAMP 10.2 software, and all values of membrane potential given throughout were corrected using this value. Current traces were sampled at 10 kHz and filtered at 5 kHz. The holding potential was −80 mV, and current-voltage relationships were elicited by 5-mV step increments to potentials between −60 mV and 60 mV for 500 ms. Peak currents were measured for each test potential, and current density was calculated by dividing peak current by cell membrane capacitance.

In patch-clamp experiments, ω-conotoxin GVIA (Alomone Labs), a specific N-type Ca^2+^ channel blocker, was used to block the N-type Ca^2+^ channel. Based on the previous study, the concentration of ω-conotoxin GVIA (1 μM) used in the present study is a saturating concentration for inhibiting N-type Ca^2+^ channels (59, 61, 62). N-type Ca^2+^ currents were obtained by subtracting Ca^2+^ currents under treatment of ω-conotoxin GVIA from total Ca^2+^ currents (59, 61).

In current-clamp experiments, AP was elicited by a ramp current injection of 0-100 pA, and the current threshold-inducing AP was measured at the beginning of the first action potential. Frequency of APs was measured in a 1-s current clamp. Input resistance was determined from the linear fit of the neuronal voltage response to hyperpolarizing current injections (20-pA step decrement from 0 to−100 pA for 1 s). The patch pipette solution was composed of (in mM): 105 K-aspartate, 20 KCl, 1 CaCl_2_, 5 MgATP, 10 HEPES, 10 EGTA, and 25 glucose (pH 7.2; 320 mOsm/L). The bath solution was composed of (in mM): 140 NaCl, 5.4 KCl, 0.5 MgCl_2_, 2.5 CaCl_2_, 5.5 HEPES, 11 glucose, and 10 sucrose (pH 7.4; 330 mOsm/L). Junction potential was calculated to be +12.3 mV, and membrane potential was corrected using this value. P-clamp 10.2 program (Axon Instruments) was used for data acquisition and analysis. All experiments were performed at room temperature (22–24°C).

### Measurement of Catalase Activity

A catalase activity assay kit (ab83464, abcam, Cambridge, UK) was used to measure the catalase activity in the AVG. The AVG was rapidly removed and washed in cold 0.01 M phosphate-buffered saline (PBS) and then homogenized on ice in assay buffer. Homogenized tissue was centrifuged (10,000 g, 4°C, 15 min), and the supernatant was transferred to a new tube. The total protein concentration in the supernatant was measured by a bicinchoninic acid protein assay kit (Cat# 23225, Thermo Fisher Scientific, Waltham, MA), and all samples were normalized to the same level of total protein concentration. Each sample (20 μl) was mixed with 58 μl of cold assay buffer in each well-followed by 12 μl of fresh 1 mM H_2_O_2_ solution into each well and incubated at 25°C for 30 min. Then 10 μl stop solution and 50 μl Developer Mix were added into each sample well and incubated at 25°C for 10 min in the dark. The OD was measured using the plate reader Infinite M200 (Tecan Group Ltd. Switzerland) at 570 nm wavelength. The corrected sample absorbance was applied to the standard curve to get the catalase activity in sample wells.

### Measurement of Cytosolic H_2_O_2_ Levels

Cytosolic H_2_O_2_ levels were measured by a mammalian expression vector encoding a fluorescence H_2_O_2_ sensor pHyPer (63, 64). Isolated CVP neurons were incubated with 1 μg/ml of pHyPer-cyto plasmid (EVN-FP941, AXXORA, Farmingdale, NY, USA) and 4 μg/ml of pn-Fect^TM^ (PN30075, Neuromics, Edina, MN, USA) for transfection. After 6-h of transfection, the medium was replaced by a mixed culture medium (a 50/50 mixture of DMEM and Ham's F12 medium supplemented with antibiotics and 10% fetal serum), and isolated CVP neurons were incubated for 48 h. The pHyPer-cyto image (green color) was captured by a Leica fluorescence microscope (Leica DMR, Leica Microsystems Inc., Buffalo Grove, IL, USA) with a digital camera (Qimaging Retiga Exi Fast 1394). The quantitative data for the fluorescence intensity in a single cell served as the cytosolic H_2_O_2_ level.

### Measurement of Intracellular Ca^2+^ Levels

Intracellular Ca^2+^ image was assessed by a calcium indicator (fluo-3/AM, F1241, Invitrogen, Carlsbad, CA, USA) and a Zeiss LSM 510 META confocal microscope with a 63 × oil immersion objective (65, 66). Isolated CVP neurons were loaded with fluo-3 (5 μM) for 40 min at 37°C in a CO2 incubator. After rinsing three times with a modified Tyrode solution (mmol/L: 140 NaCl, 5.4 KCl, 0.5 MgCl2, 2.5 CaCl2, 5.5 HEPES, 11 glucose, pH 7.4, and 330 mOsm/L), CVP neurons were placed in a recording dish with the Tyrode solution on the stage of the confocal microscope. An argon laser provided fluorescence excitation at 488 nm, and the emitted light (515 nm) was captured along with transmitted images. The Ca^2+^ fluorescent image (green color) was continuously captured every 2 s using the confocal microscope before and during high KCl (30 mM) stimulation (30 s). All analyses of intracellular Ca^2+^ levels were processed at a single-cell level, and the data was calculated by the ratio of F_Max_/F_0_, in which F_Max_ represents the fluorescence intensity at 30 s of high KCl stimulation, and F_0_ is the fluorescence intensity of the baseline (before KCl stimulation).

### Reverse–Phase Protein Microarray

Due to the limitation of small AVG samples (1–2 mg wet weight), we could not detect the expression of catalase protein using regular Western blot analyses and instead employed a modified reverse–phase protein microarray, which is highly sensitive and needs about 1 μg of protein (23, 67). AVGs were rapidly removed, immediately frozen in liquid nitrogen, and stored at −80°C until analyzed. Proteins in AVG homogenates were extracted with a lysing buffer (10 mM Tris, 1 mM EDTA, 1% SDS; pH 7.4) plus protease inhibitor cocktail (100 μl/ml, Sigma). After centrifugation at 12,000 g for 20 min at 4°C, the protein concentration in the supernatant was determined using a bicinchoninic acid protein assay kit (Pierce, Rockford, IL, USA). Fifty nanoliters of each protein sample were loaded onto nitrocellulose–coated glass slides by an 8–pin arrayer. Protein samples were then sequentially incubated with primary antibodies (rabbit anti-Catalase (D5N7V) antibody, #14097s, Cell Signaling; rabbit anti-CACNA1B (CaV2.2-α) antibody, #ACC-002, Alomone Labs; and mouse anti–β-actin antibody, Sc-4778, Santa Cruz Biotechnology) and LI–COR fluorescence–conjugated secondary antibodies (IRDye 800CW goat anti–rabbit IgG, and IRDye 680LT goat anti–mouse IgG). The protein signals were scanned with a LI–COR Odyssey IR imaging system (LI–COR, Lincoln, NE, USA).

### Statistical Analysis

All data are presented as means ± SEM. SigmaPlot 12 was used for data analysis. Statistical significance was determined by one-way or two-way ANOVA with *post-hoc* Bonferroni test for multi-group comparison. Statistical significance was determined by a Fisher exact test for incidence of ventricular arrhythmias. A student's unpaired *t*-test was used to perform a two-group comparison. Normal distribution of data was confirmed with the Kolmogorov-Smirnov test and equal variance with Levene's test. Statistical significance was accepted when p < 0.05.

## Results

### Overexpression of Catalase Attenuated T2DM-Increased Cytosolic H_2_O_2_ Levels in CVP Neurons

**Figure 1:**
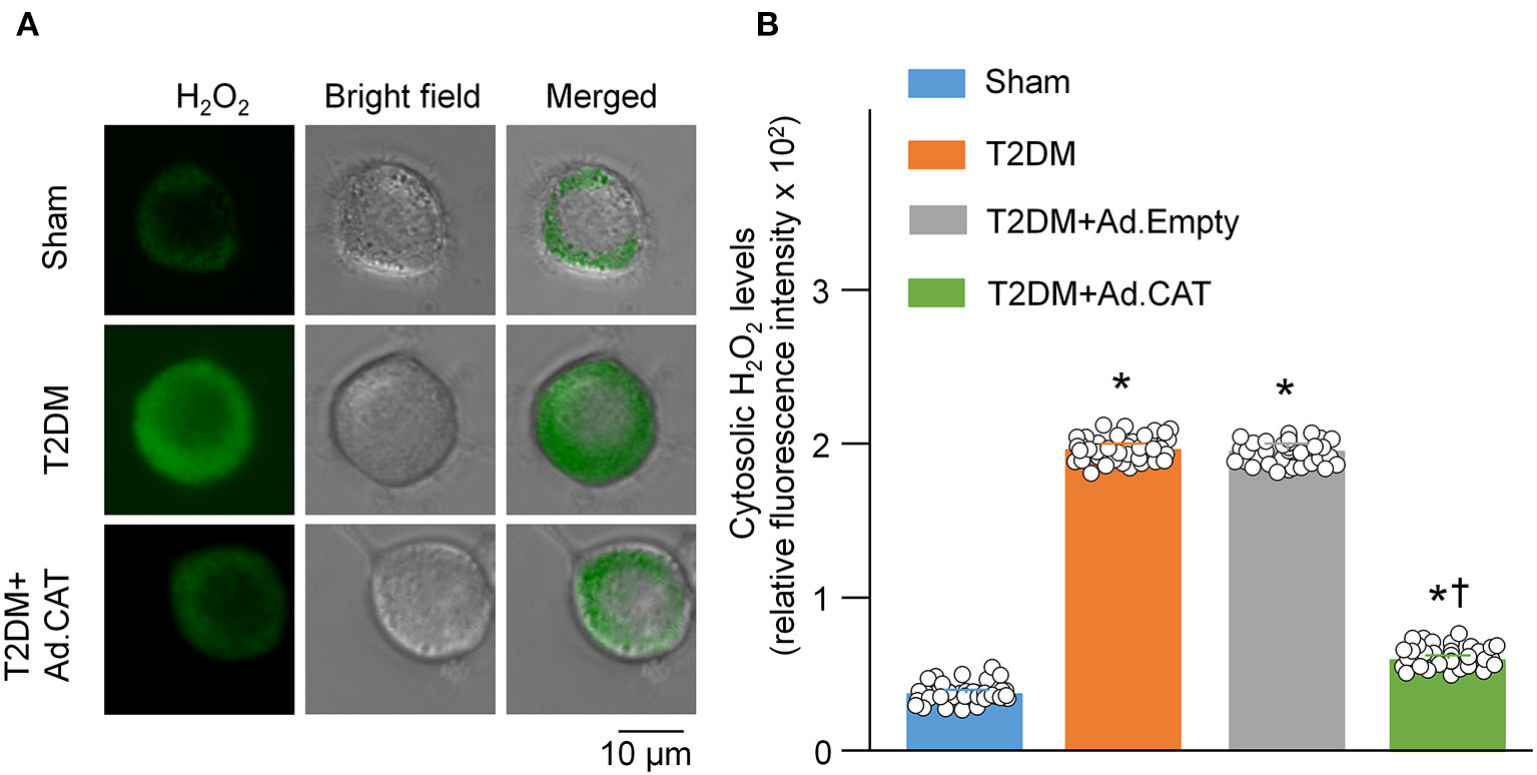
Overexpression of catalase in CVP neurons reduced intracellular H_2_O_2_ levels in T2DM rats. **(A)** Representative images of cytosolic H_2_O_2_ levels in isolated CVP neurons from sham, T2DM, and T2DM+Ad.CAT rats, measured by detecting fluorescence intensity of pHyPer-cyto (green color, a H_2_O_2_ sensor). **(B)** Quantitative data showing fluorescence intensity of pHyPer-cyto in CVP neurons in all groups. N = 40 neurons from 6 rats per group; data are means ± SEM. Statistical significance was determined by one-way ANOVA with *post-hoc* Bonferroni test. *P < 0.05 vs. sham; ^†^*P* < 0.05 vs. T2DM.

**Figure 2:**
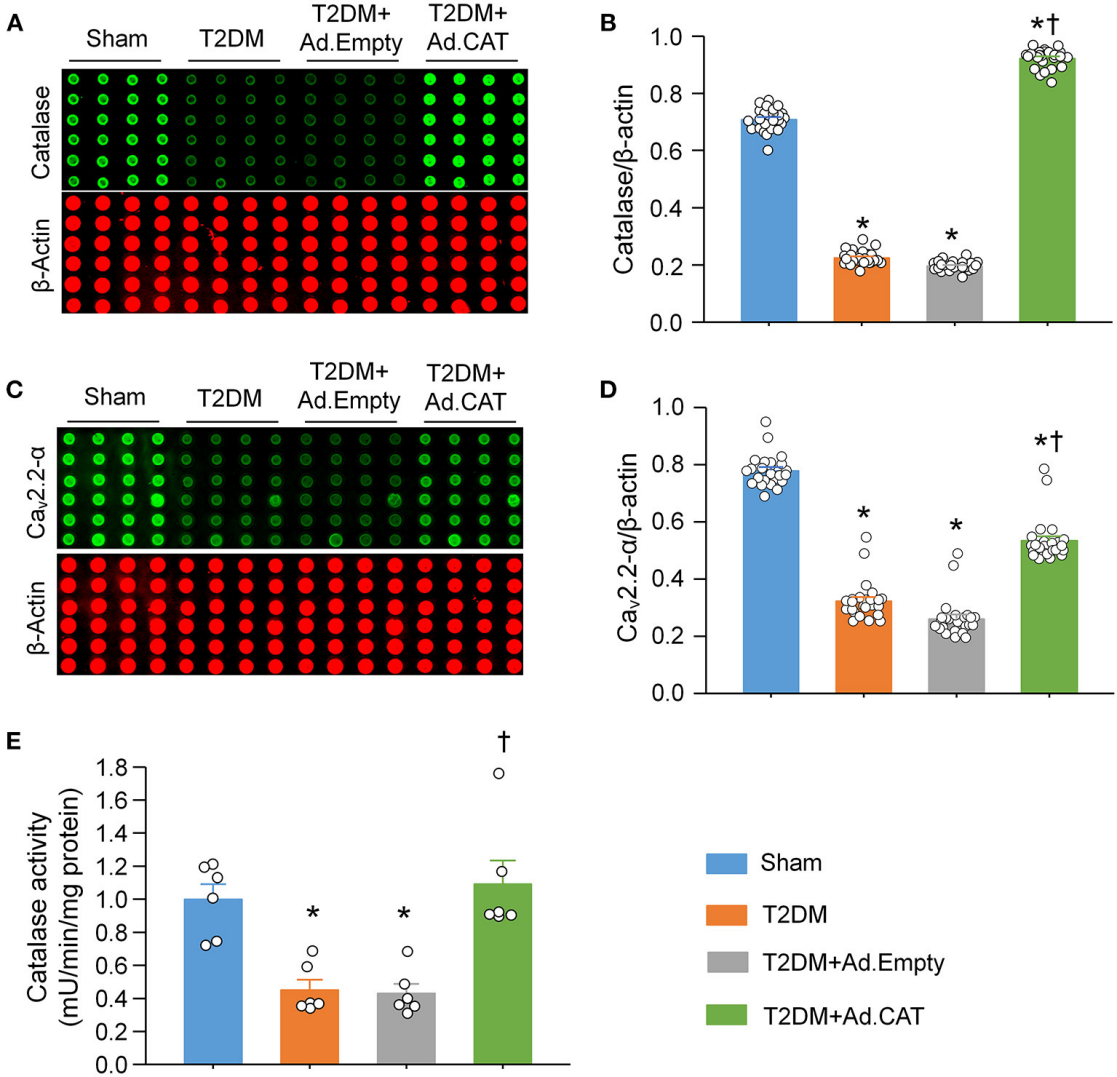
Effect of Ad.CAT gene transfection on protein expression of catalase and N-type Ca^2+^ channels (Ca_v_2.2-α) in CVP neurons in T2DM. **(A)** Raw images and **(B)** quantitative data showing protein expression of catalase in CVP neurons from all groups of rats, measured by revers–phase protein microarray. **(C)** Representative images and **(D)** quantitative data showing protein expression of Ca_v_2.2-α in the CVP neurons from all groups of rats. *N* = 24 measurements from 6 rats per group. **(E)** Catalase activity measured in all groups. *N* = 6 rats per group. Ad.CAT gene transfection significantly increased T2DM-reduced catalase activity, and protein expression of catalase and Ca_v_2.2-α in CVP neurons. Data are means ± SEM. Statistical significance was determined by one-way ANOVA with *post-hoc* Bonferroni test. *P < 0.05 vs. sham; ^†^*P* < 0.05 vs. T2DM.

Using a cytosolic H_2_O_2_ sensor pHyPer, we first compared cytosolic H_2_O_2_ levels in CVP neurons between sham and T2DM rats. Our data showed that cytosolic H_2_O_2_ levels were significantly elevated in CVP neurons from T2DM rats, compared to age-matched sham rats (195.8 ± 1.2 in the T2DM group vs. 36.5 ± 1.0 in the sham group, *P* < 0.05, Figure 1). We then measured the catalase protein (an endogenous H_2_O_2_ scavenger) and found that its expression in CVP neurons was much lower in T2DM rats than that in sham rats (0.22 ± 0.01 in the T2DM group vs. 0.71 ± 0.01 in the sham group, *P* < 0.05, Figures 2A,B). When Ad.CAT gene was microinjected into the AVG from T2DM rats, the expression of catalase protein in CVP neurons was significantly elevated (0.92 ± 0.01 in the T2DM+Ad.CAT group), compared to T2DM rats without Ad.CAT gene transfection (0.22 ± 0.01 in the T2DM group, *P* < 0.05, Figures 2A,B). Consequently, Ad.CAT gene transfection eliminated T2DM-elevated cytosolic H_2_O_2_ levels in CVP neurons (60.6 ± 1.1 in the T2DM+Ad.CAT group vs. 195.8 ± 1.2 in the T2DM group, *P* < 0.05, Figure 1). In addition, data from the measurement of catalase activity demonstrated that the catalase activity was markedly reduced in T2DM rats, compared with that in sham rats (Figure 2E). Transfection of Ad.CAT into CVP neurons totally restored T2DM-reduced catalase activity in the AVG (Figure 2E). Ad.Empty transfection failed to induce any change in cytosolic H_2_O_2_ levels, expression of catalase protein, and catalase activity in CVP neurons from T2DM rats (Figures 1, 2).

### Transfection of Ad.CAT Into CVP Neurons Improved T2DM-Reduced Protein Expression and Ion Currents of N-Type Ca^2+^ Channels, Cell Excitability, and Intracellular Ca^2+^ Levels of CVP Neurons

The reverse-phase protein microarray confirmed that protein expression of N-type Ca^2+^ channels in CVP neurons is markedly reduced in T2DM rats, compared with sham rats (0.32 ± 0.01 in the T2DM group vs. 0.78 ± 0.01 in the sham group, *P* < 0.05, Figures 2C,D), which is consistent with our previous results obtained by immunofluorescence staining (20). Ad.CAT gene transfection into CVP neurons partially restored T2DM-reduced protein expression of N-type Ca^2+^ channels (0.53 ± 0.02 in the T2DM+Ad.CAT group vs. 0.32 ± 0.01 in the T2DM group, *P* < 0.05, Figures 2C,D). Ad.Empty transfection did not affect protein expression of N-type Ca^2+^ channels (Figures 2C,D).

**Figure 3:**
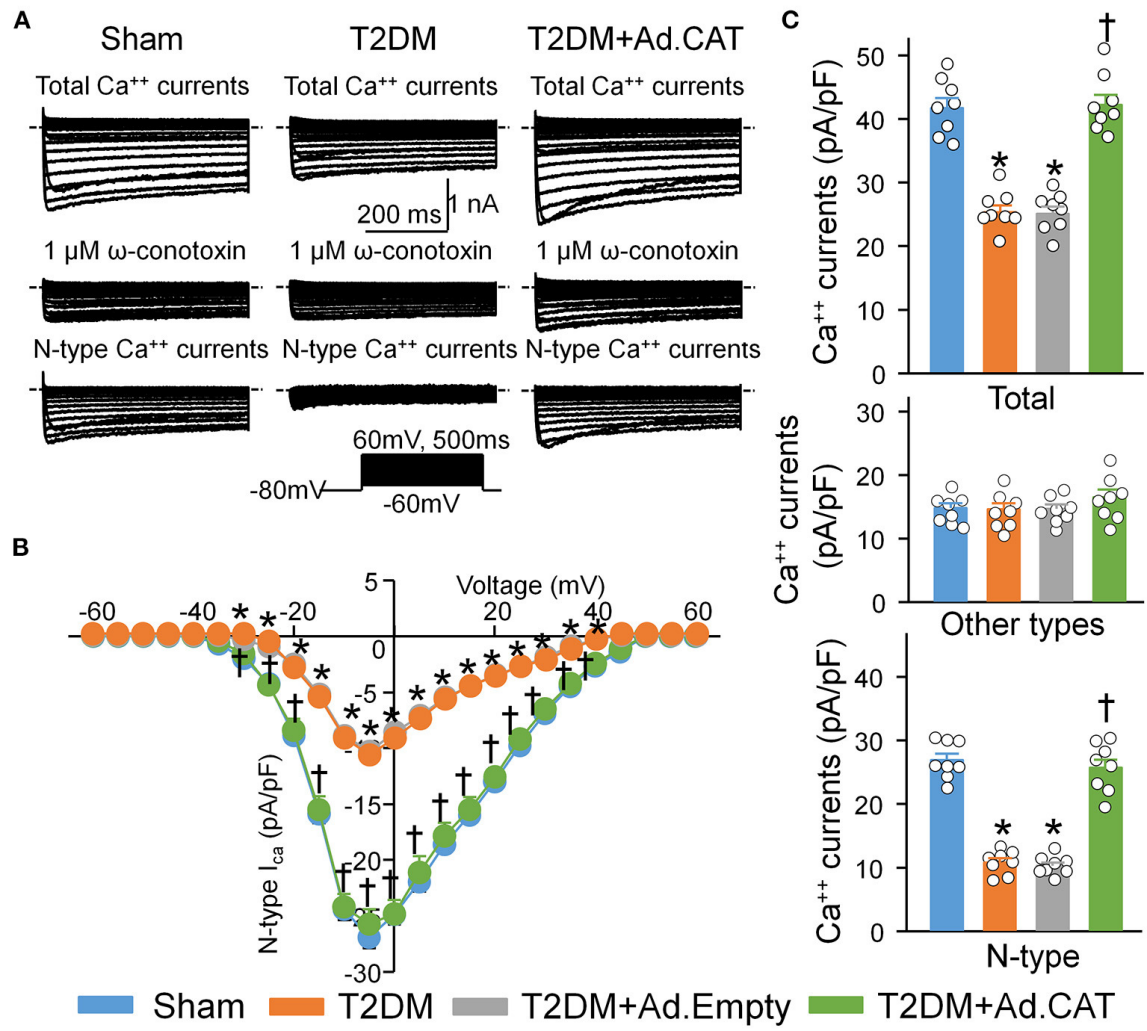
Reduction of the H_2_O_2_ levels through transfection of Ad.CAT gene increased T2DM-reduced N-type Ca^2+^ currents in CVP neurons in T2DM rats. BaCl_2_ replaced CaCl_2_ in the extracellular solution for Ca^2+^ current recording. **(A)** Original whole-cell patch-clamp recording of Ca^2+^ currents from sham, T2DM, and T2DM+Ad.CAT rats. **(B)** Current-voltage (I–V) curve of N-type Ca^2+^ currents in CVP neurons from all groups of rats. **(C)** Quantitative data of total Ca^2+^ currents, other types of Ca^2+^ currents, and N-type Ca^2+^ currents elicited by 500-ms test pulse at 0 mV from holding potential of −80 mV in CVP neurons from all groups. ω-conotoxin GVIA, a specific N-type Ca^2+^ channel blocker, was used to block the N-type Ca^2+^ channel. N-type Ca^2+^ currents were obtained by subtracting Ca^2+^ currents under treatment of ω-conotoxin GVIA from total Ca^2+^ currents. *N* = 8 neurons from 6 rats per group; data are means ± SEM. Statistical significance was determined by two-way repeated measures ANOVA with *post-hoc* Bonferroni test for data presented in **(B)**. Statistical significance was determined by one-way ANOVA with *post-hoc* Bonferroni test for data presented in **(C)**. *P < 0.05 vs. sham; ^†^*P* < 0.05 vs. T2DM.

**Figure 4:**
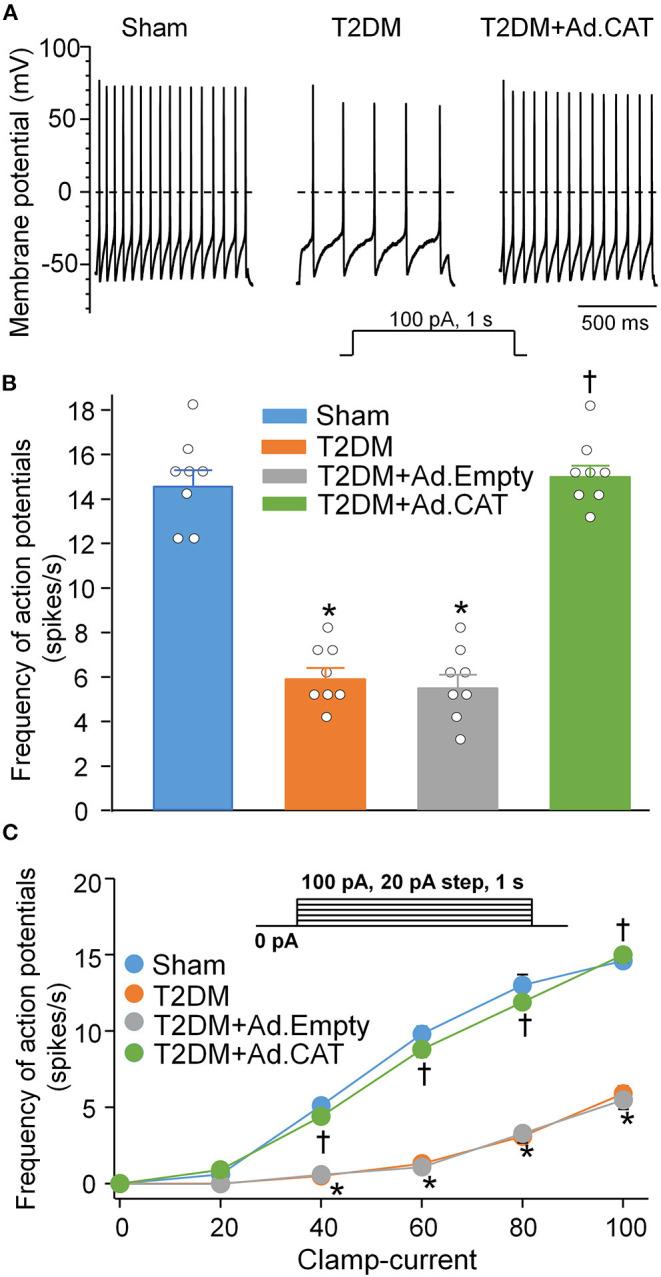
*In vivo* transfection of Ad.CAT gene restored T2DM-reduced cell excitability of CVP neurons in T2DM rats. **(A)** Original recording of action potentials (APs) in CVP neurons from sham, T2DM, and T2DM+Ad.CAT rats. **(B,C)** Quantitative data for the frequency of APs in CVP neurons from all groups. The frequency of APs was measured in a 1-s current clamp with a current injection of 100 pA. *N* = 8 neurons from 6 rats per group; data are means ± SEM. Statistical significance was determined by one-way ANOVA with *post-hoc* Bonferroni test for data presented in **(B)**. Statistical significance was determined by two-way repeated measures ANOVA with *post-hoc* Bonferroni test for data presented in **(C)**. *P < 0.05 vs. sham; ^†^*P* < 0.05 vs. T2DM.

To observe electrophysiological changes in CVP neurons, voltage-gated Ca^2+^ currents and neuronal excitability in CVP neurons were measured by the whole-cell patch-clamp technique. Our data demonstrated that total Ca^2+^ currents and cell excitability (including frequency of APs, current threshold inducing APs) of CVP neurons are markedly decreased in T2DM rats, compared with age-matched sham rats (Figure 3C, Supplementary Figure 3). Additionally, N-type Ca^2+^ currents were separated from other types of Ca^2+^ currents by treatment of ω-conotoxin GVIA, a specific N-type Ca^2+^ channel blocker (Figure 3A). Compared with sham rats (26.9 ± 1.0 pA/pF), T2DM significantly reduced N-Type Ca^2+^ currents (10.8 ± 0.7 pA/pF, *P* < 0.05, Figure 3C). However, other types of Ca^2+^ currents (i.e., Ca^2+^ currents under treatment of ω-conotoxin GVIA) were not affected by T2DM (Figure 3C). Ad.CAT gene transfection into CVP neurons significantly increased T2DM-reduced total Ca^2+^ currents (42.2 ± 1.6 pA/pF), N-type Ca^2+^ currents (25.7 ± 1.3 pA/pF), and cell excitability of CVP neurons, compared to T2DM rats without Ad.CAT gene transfection (Figures 3, 4, Supplementary Figure 3). Ad.Empty had no effects on total Ca^2+^ currents, N-type Ca^2+^ currents, and cell excitability of CVP neurons (Figures 3, 4, Supplementary Figure 3). Moreover, there were no significant differences in resting membrane potential, input resistance, and cell membrane capacitance among groups (Supplementary Table 2).

**Figure 5:**
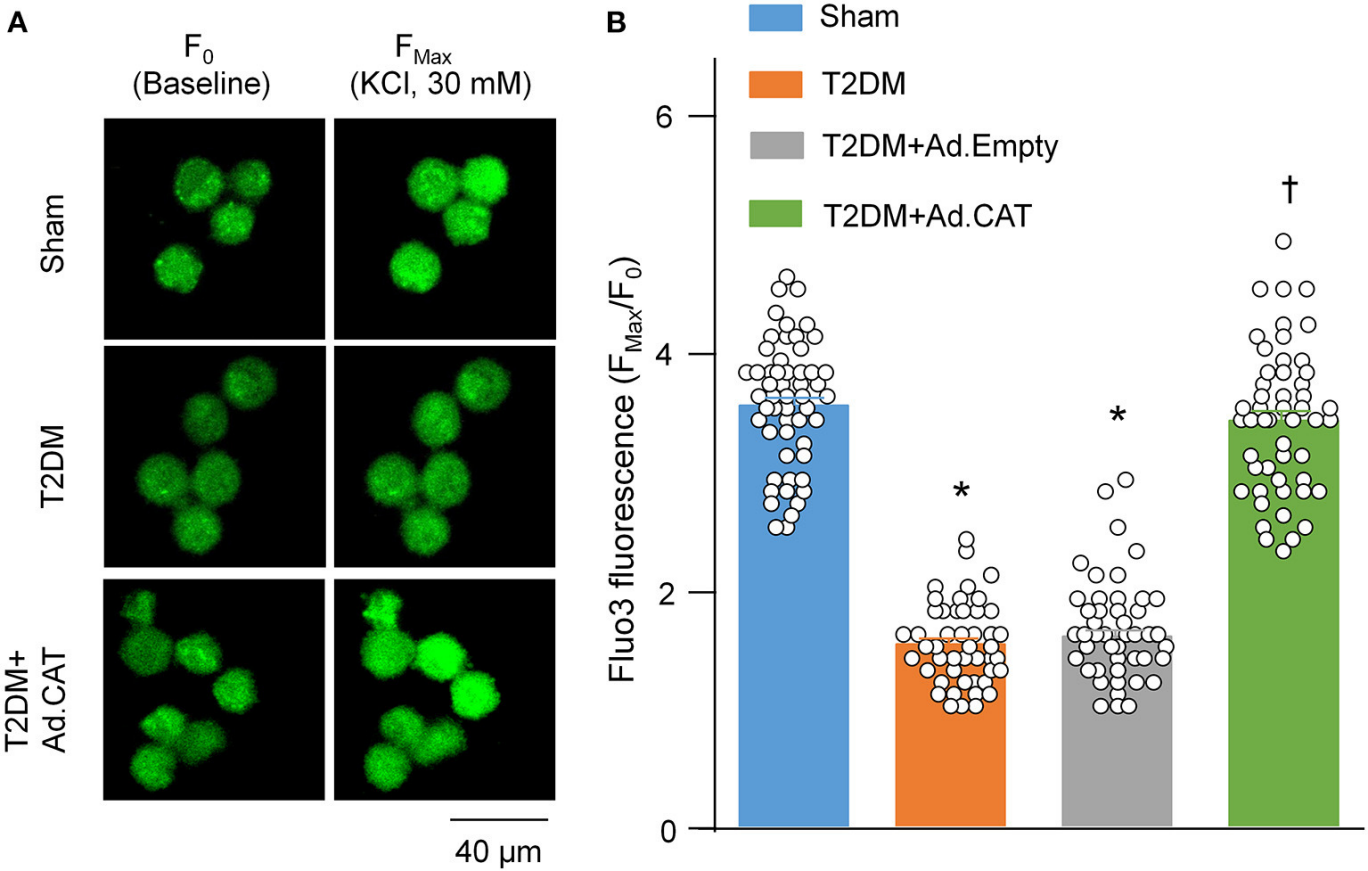
*In vivo* transfection of Ad.CAT gene restored T2DM-decreased intracellular Ca^2+^ levels in CVP neurons from T2DM rats. **(A)** Raw images of fluo-3/AM (green color, a calcium indicator) in isolated CVP neurons from sham, T2DM, and T2DM+Ad.CAT rats, in which the fluorescent image was captured by a confocal microscope before (F_0_) and during KCl (30 mM) stimulation at 30 s (F_Max_). **(B)** Mean data for the fluorescence intensity of fluo-3/AM in isolated CVP neurons from all groups of rats. *N* = 60 neurons from 6 rats per group; data are means ± SEM. Statistical significance was determined by one-way ANOVA with *post-hoc* Bonferroni test. *P < 0.05 vs. sham; ^†^*P* < 0.05 vs. T2DM.

Using Fluo3/AM with a confocal microscope, we also measured intracellular Ca^2+^ levels in all groups of rats. The ratio of F_Max_/F_0_ was significantly lower in T2DM rats than in sham rats (1.53 ± 0.04 in the T2DM group vs. 3.53 ± 0.07 in the sham group, *P* < 0.05, Figure 5). Transfection of Ad.CAT gene but not Ad.Empty into CVP neurons significantly restored intracellular Ca^2+^ levels in T2DM rats (3.39 ± 0.08 and 1.59 ± 0.05, respectively), compared to T2DM rats without adenoviral transfection (Figure 5B).

### Effect of Ad.CAT on T2DM-Reduced Vagal Control of Ventricular Function

**Figure 6:**
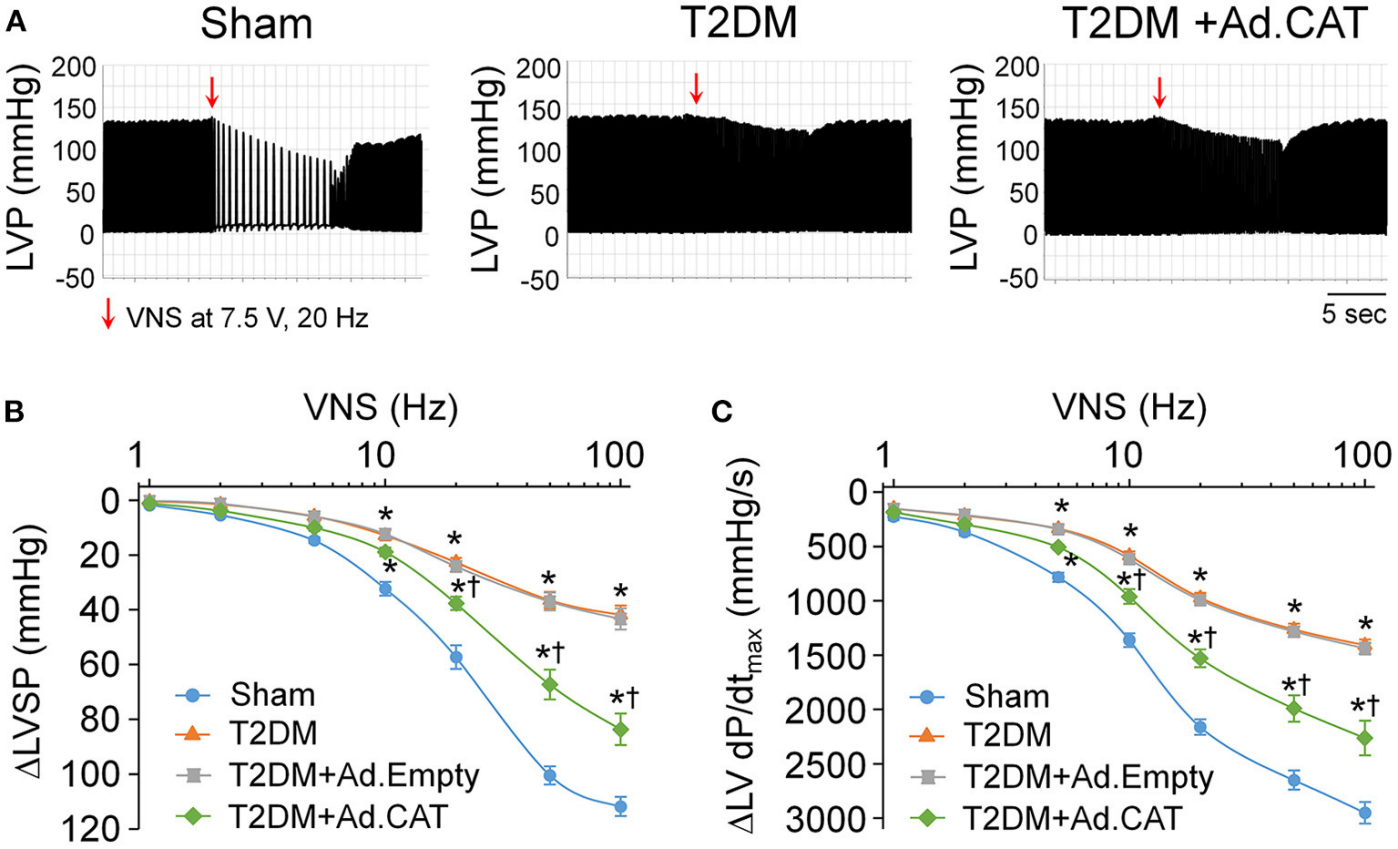
Effect of Ad.CAT gene transfection into CVP neurons on vagal control of ventricular function in T2DM rats, which was determined by changes of the left ventricular systolic pressure (LVSP) and the maximum rate of increase of left ventricular pressure (LV dP/dt_max_) in response to different frequencies of left vagal efferent nerve stimulation (VNS) in anesthetized rats. **(A)** Representative recordings demonstrating changes of the LVSP in response to 7.5 V, 20 Hz of VNS in sham, T2DM, and T2DM+Ad.CAT rats. **(B,C)** Quantitative data for changes of the LVSP **(B)** and LV dP/dt_max_ **(C)** in response to different frequencies (1–100 Hz) of VNS in all groups of rats. *In vivo* Ad.CAT gene transfection into CVP neurons significantly improved T2DM-blunted vagal control of ventricular function. *N* = 6 rats per group; data are means ± SEM. Statistical significance was determined by two-way repeated measures ANOVA with *post-hoc* Bonferroni test. *P < 0.05 vs. sham; ^†^*P* < 0.05 vs. T2DM.

The vagal control of ventricular function, an index of ventricular vagal function, was evaluated by detecting changes of LVSP and LV dP/dt_max_ in response to different frequencies of VNS in anesthetized rats. Compared to age-matched sham rats, changes of LVSP and LV dP/dt_max_ in response to different frequencies (2–100 Hz) of VNS were blunted in T2DM rats (Figure 6). These data indicated that the ventricular vagal function was impaired in the T2DM state. Ad.CAT gene transfection into CVP neurons partially improved T2DM-reduced ventricular vagal function, as evidenced by improved responses of LVSP and LV dP/dt_max_ to different frequencies of VNS in T2DM+Ad.CAT rats (Figure 6). Ad. Empty transfection into CVP neurons failed to ameliorate ventricular vagal function in T2DM rats (Figures 6B,C).

### Effect of Ad.CAT on T2DM-Induced Cardiac Autonomic Dysfunction in Conscious Rats

**Figure 7:**
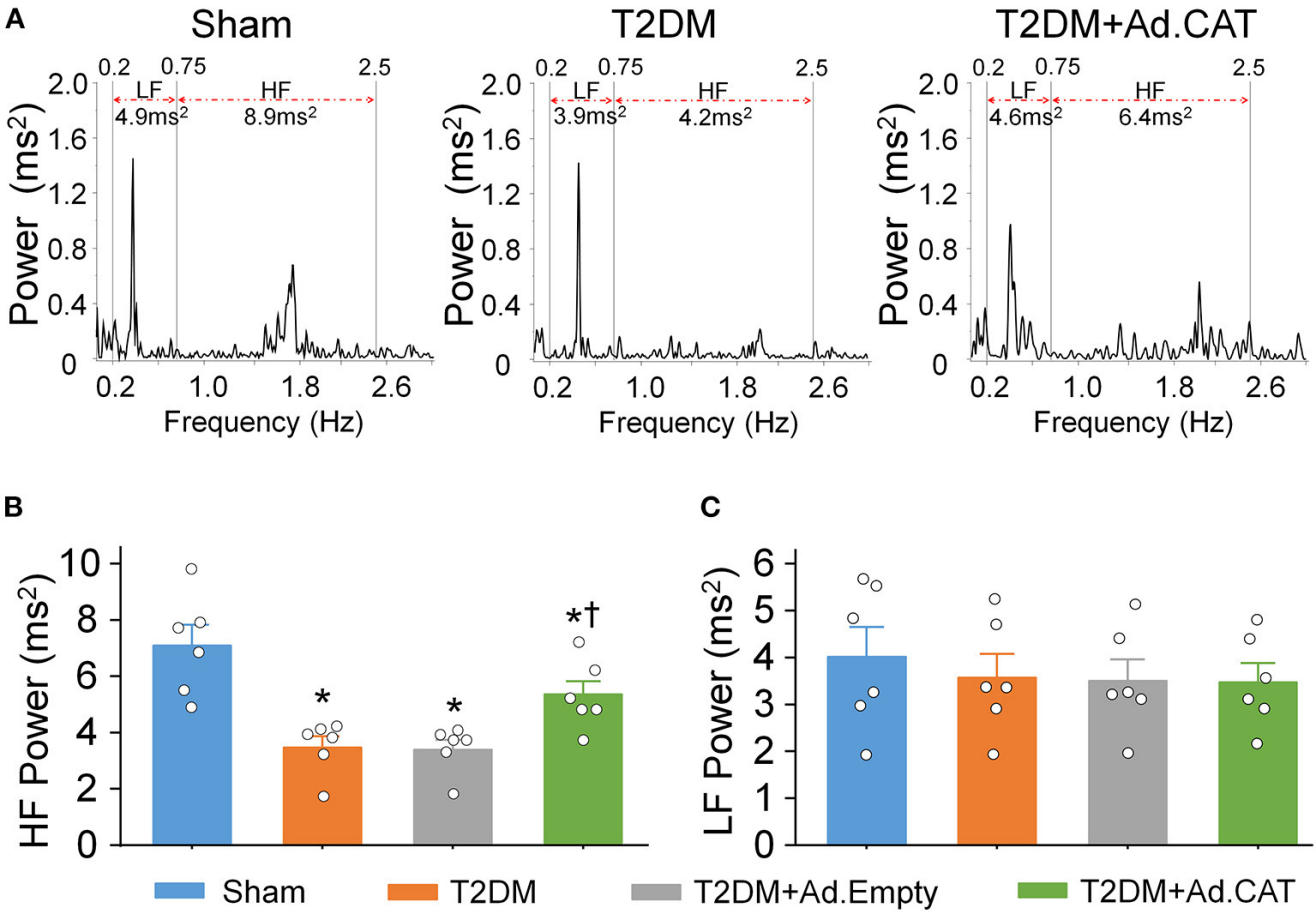
*In vivo* transfection of Ad.CAT gene into CVP neurons improved T2DM-attenuated cardiac vagal activation, measured by the power spectral analysis of heart rate variability (HRV) in conscious rats. **(A)** Representative tracings of HRV analyzed from 24-h ECG recording in sham, T2DM, and T2DM+Ad.CAT rats. Spectral power was quantified for LF from 0.2 to 0.75 Hz and HF from 0.75 to 2.5 Hz. **(B,C)** Quantitative data of HF **(B)** and LF **(C)** from all groups of conscious rats. *N* = 6 rats per group; data are means ± SEM. Statistical significance was determined by one-way ANOVA with *post-hoc* Bonferroni test. *P < 0.05 vs. sham; ^†^*P* < 0.05 vs. T2DM.

Autonomic dysfunction, including cardiac sympathetic and parasympathetic imbalances, is a common complication in T2DM patients (68–70). Given that HRV analysis is the most common method for the diagnosis of cardiac autonomic dysfunction in T2DM patients (49, 50), it was employed to evaluate the cardiac autonomic function from a 24-h continuous ECG recording in conscious rats. Data from the power spectral analysis of the HRV demonstrated that HF power (an index of cardiac parasympathetic activation) was significantly reduced, whereas the LF power (a marker of cardiac sympathetic activation) was slightly reduced in T2DM rats, compared with sham rats (Figure 7). These data are consistent with results from our recent publication (24) and one clinical report that vagal predominance was significantly impaired in proportion to a withdrawal of total autonomic activity (71). Ad.CAT gene transfection into CVP neurons partially restored T2DM-impaired cardiac vagal activation recorded in T2DM+Ad.CAT conscious rats (Figures 7A,B).

### Transfection of Ad.CAT Into CVP Neurons Alleviated the Heterogeneity of Ventricular Electrical Activity in Conscious T2DM Rats

**Figure 8:**
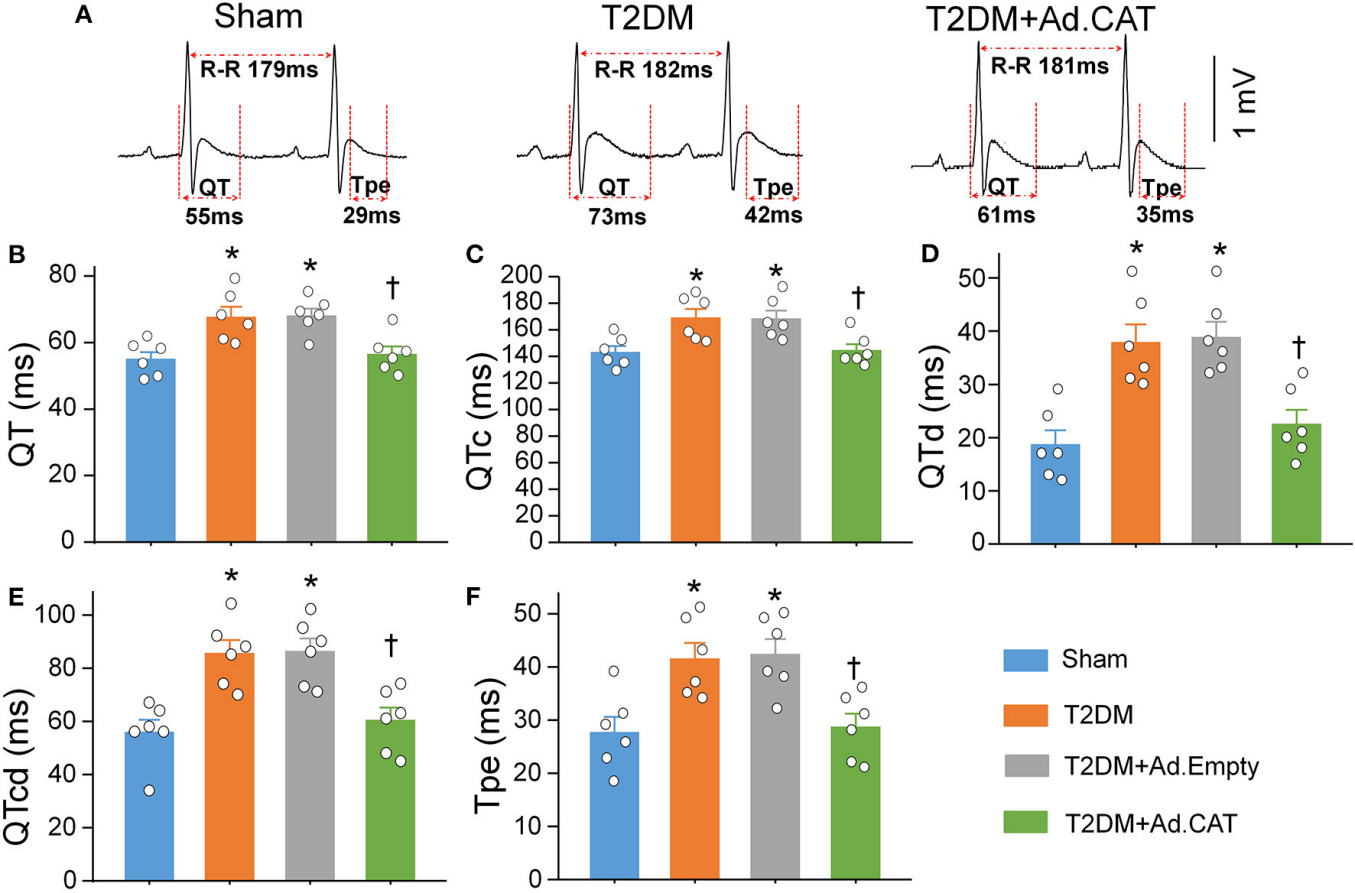
Reduction of intracellular H_2_O_2_ levels in CVP neurons attenuated the heterogeneity of ventricular electrical activities in conscious T2DM rats. **(A)** Representative tracings for QT and Tpe intervals in sham, T2DM, and T2DM+Ad.CAT rats. **(B–F)** Quantitative data for QT interval **(B)**, QTc interval **(C)**, QT dispersion **(D)**, QTc dispersion **(E)**, and Tpe **(F)** in all experimental groups. *N* = 6 rats per group; data are means ± SEM. Statistical significance was determined by one-way ANOVA with *post-hoc* Bonferroni test. *P < 0.05 vs. sham; ^†^*P* < 0.05 vs. T2DM.

Since the heterogeneity of ventricular electrical activity is a critical factor of ventricular arrhythmogenesis (48), ventricular arrhythmogenesis-related ECG markers, including QT and QTc intervals, QT and QTc dispersions, and Tpe interval, were also calculated from 24-h continuous ECG recording. QT and QTc intervals, QT and QTc dispersions, and Tpe interval were significantly elongated in T2DM rats, compared with sham rats (Figure 8), which suggest that T2DM increased the spatial and transmural dispersion of ventricular repolarization. Ad.CAT gene but not Ad.Empty transfection into CVP neurons markedly reduced T2DM-increased heterogeneity of ventricular electrical activity, as demonstrated by significant shorting in QT and QTc intervals, QT and QTc dispersions, and Tpe interval in the T2DM+Ad.CAT group toward the levels in the sham group (Figures 8A–F).

### Transfection of Ad.CAT Into CVP Neurons Reduced the Susceptibility to Ventricular Arrhythmias in Anesthetized T2DM Rats

**Figure 9:**
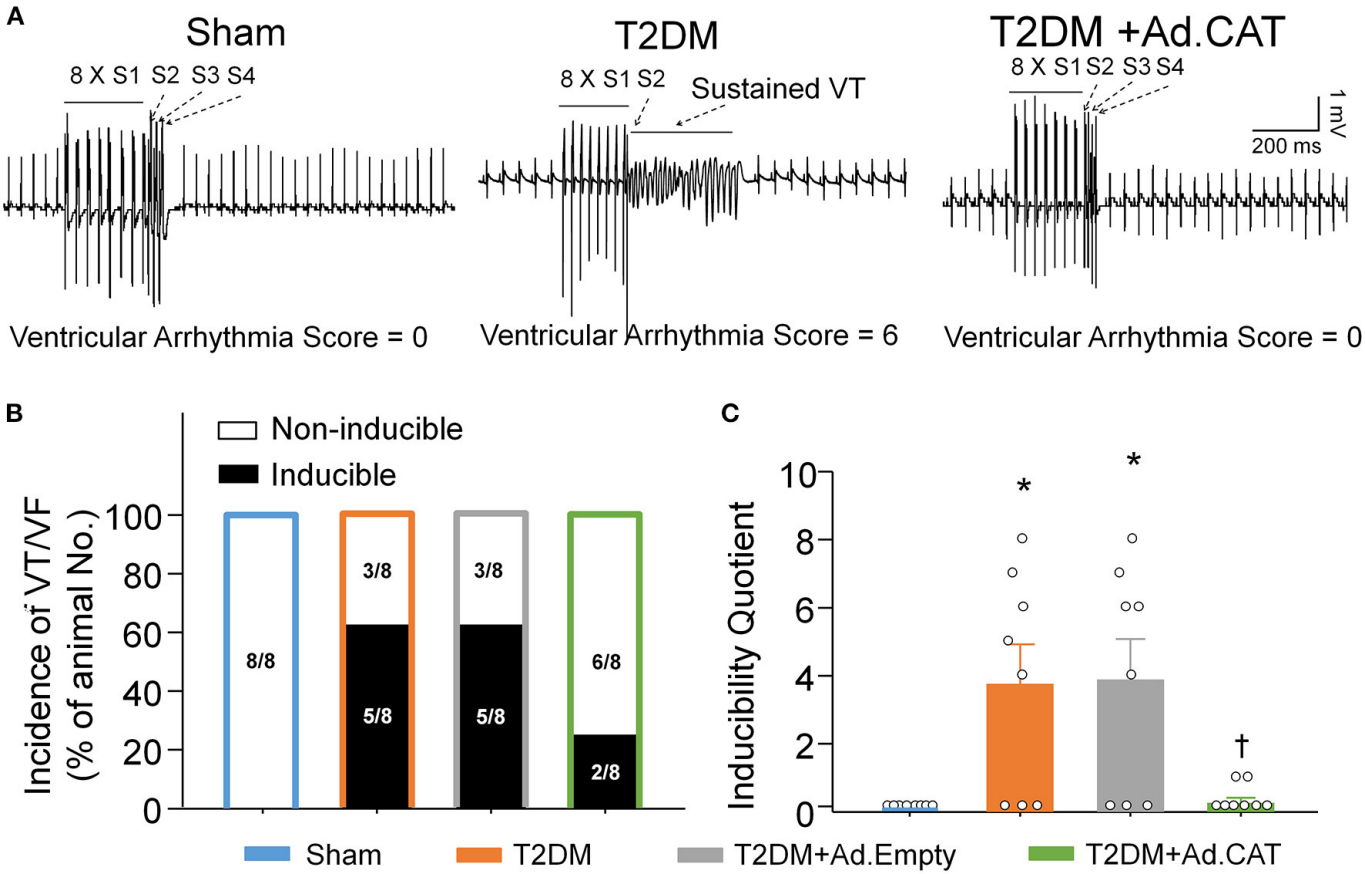
Effect of Ad.CAT gene transfection into CVP neurons on susceptibility to ventricular tachyarrhythmia in anesthetized T2DM rats. **(A)** Raw data for PES-evoked VT/VF in anesthetized sham, T2DM, and T2DM+Ad.CAT rats. **(B,C)** Mean data for incidence **(B)** and inducibility quotient **(C)** of PES-evoked VT/VF in all groups of rats. Ad.CAT gene transfection into CVP neurons markedly decreased the inducibility quotient of PES-evoked VT/VF in T2DM rats. *N* = 8 rats per group; data are means ± SEM. Statistical significance was determined by Fisher exact test for the incidence of VT/VF and one-way ANOVA with *post-hoc* Bonferroni test for inducibility quotient of VT/VF. *P < 0.05 vs. sham; ^†^*P* < 0.05 vs. T2DM.

PES-triggered inducibility of ventricular arrhythmias was used to evaluate ventricular arrhythmogenesis in all experimental groups of anesthetized rats. As demonstrated in Figure 9, PES failed to elicit the occurrence of VT/VF and the inducibility quotient was zero in sham rats. In T2DM rats, PES induced VT/VF with a high incidence (63%) and inducibility quotient (3.75 ± 1.18), compared to age-matched sham rats (Figures 9B,C). Ad.CAT gene transfection into CVP neurons failed to induce the significant reduction in the incidence of VT/VF, but it significantly reduced the susceptibility to ventricular arrhythmias (0.38 ± 0.26 for the inducibility quotient) in T2DM rats (Figures 9A–C). Ad.Empty transfection in CVP neurons did not affect the susceptibility to ventricular arrhythmias in anesthetized T2DM rats (Figures 9B,C).

### Transfection of Ad.CAT Into CVP Neurons Mitigated MI-Induced Ventricular Arrhythmias in Conscious T2DM Rats

**Figure 10:**
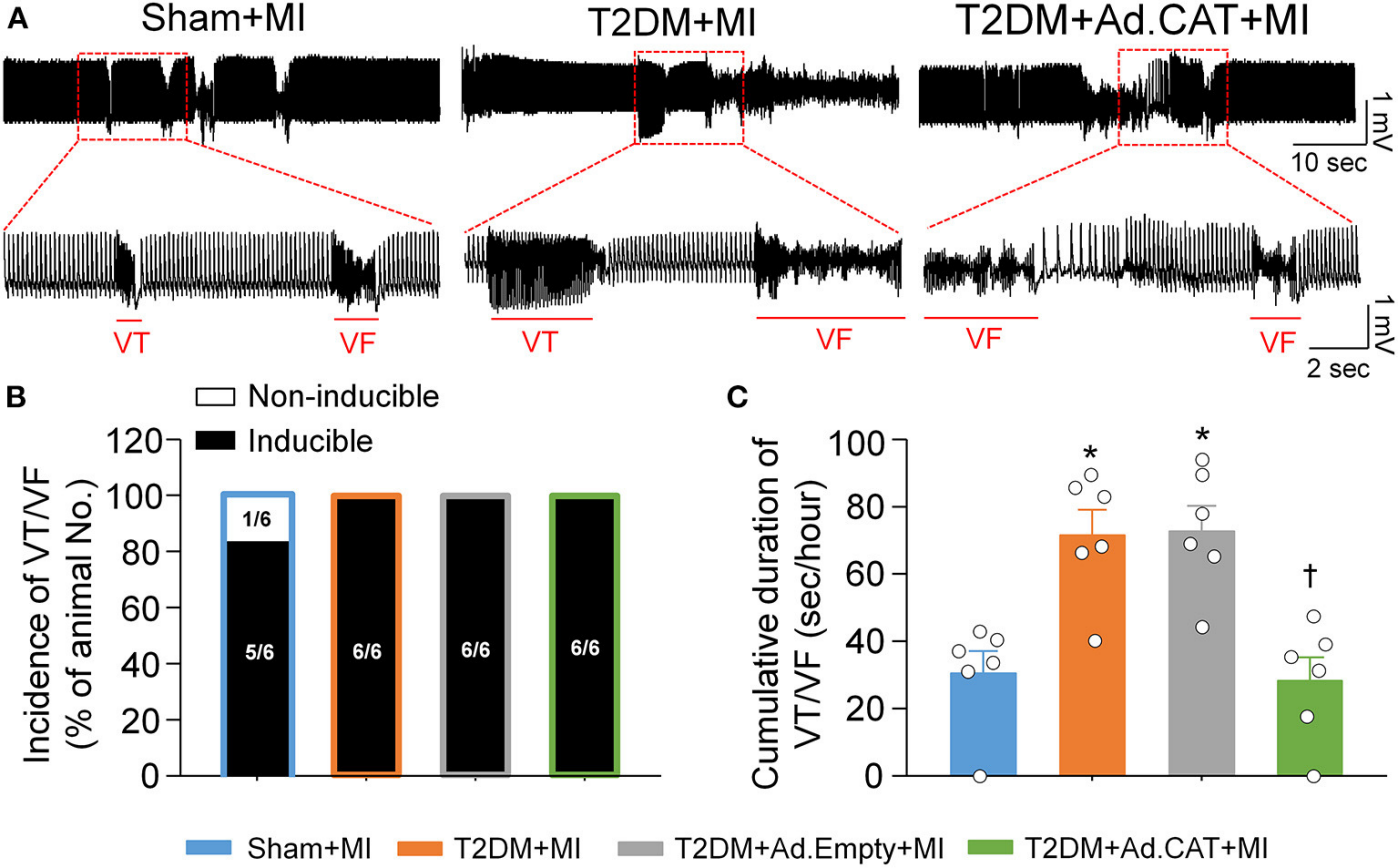
*In vivo* transfection of Ad.CAT gene into CVP neurons suppressed myocardial infarction (MI)-evoked ventricular arrhythmias in conscious T2DM rats. **(A)** Raw ECG recordings of VT/VF in conscious sham, T2DM, and T2DM+Ad.CAT rats. **(B,C)** Mean data for incidence **(B)** and cumulative duration of VT/VF **(C)** in all groups of conscious rats. *N* = 6 rats per group; data are means ± SEM. Statistical significances in the incidence of VT/VF and cumulative duration of VT/VF were determined by Fisher exact test and one-way ANOVA with *post-hoc* Bonferroni test, respectively. *P < 0.05 vs. sham; ^†^*P* < 0.05 vs. T2DM.

Acute MI-induced ventricular arrhythmic events such as VT/VF were compared in all experimental groups of conscious rats. In T2DM rats, MI induced VT/VF with a long cumulative duration (71.7 ± 7.4 s/h), compared to the sham+MI group (30.7 ± 6.4 s/h; *P* < 0.05, Figure 10). Ad.CAT gene transfection into CVP neurons markedly reduced the cumulative duration of VT/VF, whereas it failed to lower the incidence of VT/VF induced by MI in T2DM rats (28.3 ± 6.9 s/h and 100%, respectively, Figure 10). Ad.Empty transfection into CVP neurons had no effect on MI-evoked ventricular arrhythmias in T2DM rats (Figures 10B,C).

## Discussion

### Major Findings

In the present study, we reported a major contribution of H_2_O_2_-N-type Ca^2+^ channel signaling pathway to the withdrawal of cardiac vagal activity and ventricular arrhythmogenesis in the T2DM state. We demonstrated for the first time that endogenous H_2_O_2_ elevation was involved in T2DM-decreased protein expression and ion currents of N-type Ca^2+^ channels, intracellular Ca^2+^ levels, and cell excitability in CVP neurons. *In vivo* transfection of Ad.CAT gene into CVP neurons normalized H_2_O_2_ levels, elevated N-type Ca^2+^ channel activation and neuronal excitability, and improved impaired cardiac vagal activity in T2DM rats. Additionally, Ad.CAT gene transfection also restored the heterogeneity of ventricular electrical activities, reduced the susceptibility to ventricular arrhythmias, and suppressed acute MI-evoked ventricular arrhythmias in T2DM rats. The direct evidence from our present study clarified the contribution of endogenous H_2_O_2_ elevation to the CVP neuronal dysfunction, and consequent withdrawal of cardiac vagal activity and ventricular arrhythmogenesis in the T2DM state.

### Endogenous H_2_O_2_ Elevation in the T2DM State

Reactive oxygen species (ROS), such as superoxide, H_2_O_2_, and hydroxyl radical, have an important role in regulations of the physiological and pathophysiological signal transduction (72). ROS are produced from numerous non-enzymatic (73) and enzymatic reactions in various cell compartments such as mitochondria, cytoplasm, cell membrane, and endoplasmic reticulum, etc. (72) during mitochondrial oxidative metabolisms and in the cellular response to cytokines, bacterial invasion, and xenobiotics (74). At the physiological condition, a low level of ROS is essential for the maintenance of physiological functions, including the cell proliferation, migration, differentiation (27, 28), signaling transduction, and gene expression (29). However, excessive ROS production can damage the cellular macromolecules and supramolecular complexes and activate specific signaling pathways (30), leading to the pathogenesis of T2DM (31, 32). Among the members of the ROS, H_2_O_2_ and superoxide have been the main investigative foci of ROS biology in recent years (75). Since H_2_O_2_ is a relatively stable ROS (25, 26), we focused on the involvement of H_2_O_2_ in the CVP neuronal dysfunction, withdrawal of cardiac vagal activity and ventricular arrhythmogenesis in the T2DM state in the current study. Although our study demonstrated that intracellular H_2_O_2_ levels in CVP neurons are significantly increased in T2DM rats (Figure 1), the source of H_2_O_2_ and mechanisms responsible for the intracellular H_2_O_2_ elevation in CVP neurons remain unclear. Among the several sources of the ROS, the mitochondrial electron transport chain is thought to be an essential pathway to produce the ROS in the T2DM state (32, 76). It has been reported that the electron transport chain is activated by chronic hyperglycemia, which leads to the production of more significant amounts of the ROS and subsequent deterioration of β-cell function in T2DM (32). In addition, our current study demonstrated that catalase activity and expression of catalase protein in AVG were markedly reduced in T2DM (Figure 2), which might be another reason for T2DM-elevated endogenous H_2_O_2_ levels because catalase serves as an endogenous H_2_O_2_ scavenger. However, it remains unclear how T2DM reduced catalase activity and expression of catalase protein in AVG. Future studies are needed to explore the mechanisms associated with T2DM-elevated H_2_O_2_ levels in CVP neurons.

### Endogenous H_2_O_2_ Elevation Impaired N-Type Ca^2+^ Channel Function in CVP Neurons in T2DM

Accumulating evidence has shown that H_2_O_2_ modulates the cellular function through regulating ion channels such as voltage-gated Ca^2+^ channels (36–38) and potassium (K^+^) channels (77, 78) in various tissues and cells. There are five types of Ca^2+^ channels, including L-, T-, N-, R-, and P/Q-type channels, that have been functionally characterized in central and peripheral neurons (79, 80). Among the various types of voltage-gated Ca^2+^ channels, N-type Ca^2+^ channels, predominantly expressed in the nervous system, play an important role in modulation of the neurotransmitter release at nerve terminals (81, 82). Our previous study demonstrated that T2DM only reduced the mRNA and protein expression of N-type Ca^2+^ channels, rather than other types of Ca^2+^ channels (including L-, N-, P/Q-, and R-type Ca^2+^ channels) in CVP neurons (20). In the present study, we reported the similar results that T2DM decreased N-type Ca^2+^ currents but not other types of Ca^2+^ currents in CVP neurons (Figure 3C). Simultaneously, T2DM also reduced intracellular Ca^2+^ levels and cell excitability of CVP neurons (Figures 4, 5). When Ad.CAT gene transfection into CVP neurons increased over the expression and activity of catalase, decreased intracellular H_2_O_2_ levels, restored protein expression and ion currents of N-type Ca^2+^ channels, and increased intracellular Ca^2+^ levels and cell excitability of CVP neurons in T2DM rats (Figures 1–5), we believe that intracellular H_2_O_2_ elevation causes the reduction of N-type Ca^2+^ channel expression and activation in CVP neurons and the latter decreases intracellular Ca^2+^ levels and cell excitability of CVP neurons in T2DM rats.

It has been widely reported that transient exposure to exogenous H_2_O_2_ augments cytosolic Ca^2+^ levels through multiple voltage-gated Ca^2+^ channels in various cells (36–38, 83, 84), which is not consistent with the data in our present study (Figure 5). There are several possibilities for this discrepancy as discussed below. First, modulating ion channel function includes acute changes of ion channel kinetics and chronic alterations of the ion channel expression. Being different from studies mentioned above, we isolated primary CPV neurons from sham and T2DM animals to test the regulatory role of endogenous H_2_O_2_ in both the maximal current amplitude and protein expression of N-type Ca^2+^ channels (Figures 2–4). The integrative effects of endogenous H_2_O_2_ elevation on both the maximal current amplitude and protein expression of N-type Ca^2+^ channels reduced intracellular Ca^2+^ levels in CVP neurons from T2DM rats (Figure 5). Second, T2DM-induced endogenous H_2_O_2_ elevation differs from exogenously applied H_2_O_2_ concentration. Third, there are different responses of Ca^2+^ channels to exogenously applied H_2_O_2_ in different cells, because Whyte et al. reported that H_2_O_2_ inhibits total Ca^2+^ currents and cell excitability of CVP neurons when H_2_O_2_ was transiently applied in isolated CVP neurons from normal rats (85).

Superoxide also is one of the ROS in many tissues during diabetes (35). It is very difficult to distinguish the signaling pathway between H_2_O_2_ and superoxide, considering that superoxide is rapidly dismutated into H_2_O_2_ by superoxide dismutase (86–88). In our present study, Ad.CAT gene transfection into CVP neurons decreased intracellular H_2_O_2_ levels, enhanced protein expression and ion currents of N-type Ca^2+^ channels, and increased intracellular Ca^2+^ levels and cell excitability of CVP neurons in T2DM rats (Figures 1–5), which confirms that H_2_O_2_ is the primary trigger to induce changes of these variables in CPV neurons from T2DM rats.

### Dysfunction of N-Type Ca^2+^ Channels in CVP Neurons Partially Contributed to T2DM-Induced Withdrawal of Cardiac Vagal Function

Traditional teaching has stated that cardiac vagal nerves slow sinus rate and atrioventricular conduction, with little influence on the ventricle. This is because ventricular vagal innervation was considered sparse in historical reports. However, newer histological techniques have challenged this traditional principle and affirmed dense vagal innervation in the ventricle from all species including mouse, rat, cat, dog, pig, sheep, and human (89–95). Our previous study has confirmed the relationship between cardiac vagal function (in the AVG) and the contractile and electrophysiological function of ventricles in sham rats (23). Our current study confirmed that vagal nerve stimulation induced significant reduction in LVSP, confirming the functional innervation of the vagal nerve in the left ventricle. Ad. CAT-induced improvements in the LVSP, QTc and QTd might be achieved by restoring cell excitability of CVP neurons, which subsequently increases ACh release from the vagal nerve terminals, improves vagal control of ventricular function and ventricular electrophysiological activities. Detailed mechanisms are needed to explore in future.

T2DM-induced impairments in the vagal control of ventricular function (Figure 6), and cardiac vagal activation (Figure 7) are significantly but not fully restored by *in vivo* Ad.CAT gene transfection. Since Ad.CAT gene transfection into the AVG normalized T2DM-attenuated N-type Ca^2+^ currents and cell excitability of CVP neurons toward the levels in sham rats (Figures 3, 4), the present study suggests that CVP neuronal dysfunction partially contributes to T2DM-impaired cardiac vagal function. T2DM-induced impairments of other components, including pre-synaptic elements such as ACh release from vagal nerve terminals, and post-synaptic components such as muscarinic ACh receptors (mAChRs) and responses of cardiac myocytes to the cardiac vagal activation, may be involved in T2DM-impaired cardiac vagal function. One previous study reported that ACh release from cardiac vagal nerve terminals was decreased in T2DM patients (19), although the distribution of cardiac vagal nerve terminals remains unclear in the T2DM state. Additionally, protein expression of type-2 mAChRs is downregulated in left ventricle from T2DM mouse, whereas it was unchanged in left ventricle samples from T2DM patients with coronary artery disease (96). It is possible that coronary artery disease affects protein expression of mAChRs in left ventricles from T2DM patients with coronary artery disease. Moreover, diabetic cardiomyopathy characterized by structural and functional abnormalities in the ventricle (97) might contribute to T2DM-impaired cardiac vagal function. Further studies are required to address if and how these components are involved in cardiac vagal dysfunction in T2DM.

### Targeting H_2_O_2_-N-Type Ca^2+^ Channel Signaling Pathway in CVP Neurons Is an Effective Intervention Against MI-Evoked Ventricular Arrhythmias in T2DM

The leading cause of mortality and morbidity in patients with T2DM is cardiovascular diseases (98, 99), among which acute MI-related ventricular arrhythmia is the primary cause of mortality in T2DM (3, 6). Patients with T2DM are two to four times more likely to die from MI than non-diabetic patients (6, 100). Our recent study found that the decrease in cell excitability of CVP neurons contributes to the withdrawal of cardiac vagal activity and MI-evoked ventricular arrhythmias and high mortality in T2DM rats (24). Our current study further demonstrated that endogenous H_2_O_2_ elevation is a critical factor for dysfunction of CVP neurons and subsequent impairment of the cardiac vagal activity in the T2DM state. Although catalase (a H_2_O_2_ scavenger) has generated intense interest as an antioxidant therapy, a short half-life, poor cellular uptake, and inability to safely and efficiently deliver catalase into tissues significantly limit its therapeutic use (101). For the first time, we successfully attenuated the oxidative stress through *in vivo* gene transfection of Ad.CAT into CVP neurons in T2DM rats, as evidenced by an increase in catalase activity, protein expression of catalase, and a subsequent decrease in H_2_O_2_ levels in CVP neurons at 1 week after Ad.CAT gene transfection (Figures 1, 2). *In vivo* Ad.CAT gene transfection into CVP neurons markedly improved the heterogeneity of ventricular electrical activity, reduced the susceptibility to ventricular arrhythmias, and suppressed MI-evoked ventricular arrhythmias in T2DM rats. In addition, our previous study has already demonstrated that gene knock-down of N-type Ca^2+^ channels in AVG markedly attenuates vagal control of ventricular function and increases the susceptibility to ventricular arrhythmias by attenuating cell excitability of AVG neurons. Our current study further confirmed that restoration of the N-type Ca^2+^ channel expression by Ad.CAT gene transfection into AVG successfully improves the cell excitability of AVG neurons, ventricular vagal function, and ventricular arrhythmogenesis in T2DM rats. These data suggested that N-type Ca^2+^ channels are the major contributor to the beneficial effects observed in current study. Based on these data, we believe that targeting H_2_O_2_-N-type Ca^2+^ channel signaling pathway in CVP neurons is an effective intervention against MI-evoked ventricular arrhythmias in the T2DM state. Of course, we cannot rule out the involvement of myocardial remodeling in MI-evoked malignant ventricular arrhythmias in T2DM because much evidence has shown that the remodeling of ion channels and action potential duration in ventricular myocytes from diabetic heart is associated with ventricular arrhythmias (102–105).

## Study Limitations and Perspectives

First, the HRV is a commonly used method for determination of the autonomic function in T2DM patients in the clinic (49, 50). Although it predominantly provides the information about the autonomic innervation of the sinoatrial node (106), we cannot rule out its relevance with the autonomic innervation in the ventricle because it measures the specific changes in variability between successive R–R intervals (ventricular beats). It has become the conventionally accepted conception to describe variations of both instantaneous heart rate and R–R intervals (107). We believe that the HRV represents the autonomic innervation not only in the sinoatrial node but also the ventricle. However, it is impossible to distinguish the autonomic innervation between the sinoatrial node and ventricle by the power spectral analysis of the HRV, which will require the development of advanced techniques not yet available. Second, short–term variability of the QT interval (STVQT), an important electrophysiological marker, is widely used in clinic for predicting ventricular arrhythmias (108–110). However, we did not calculate the STVQT in our current study, because it is hard to do manually from 24-h ECG recordings and the Labchart 8 software (AD Instruments) used to analyze the ECG and HRV in our laboratory does not equip with the function of the STVQT calculation. We will request the function of the STVQT calculation from the AD Instruments for our future studies. Third, our current study found that endogenous H_2_O_2_ affects expression of N-type Ca^2+^ channels in CVP neurons. However, the mechanisms for the effect of endogenous H_2_O_2_ on expression of N-type Ca^2+^ channels are unclear. Additionally, an electrophysiological study in isolated rat cardiac vagal neurons has shown that exogenous H_2_O_2_ acutely inhibits voltage-gated calcium channels and decreases cell excitability (85). Therefore, the potential mechanisms including some intracellular signaling pathways and direct influence of endogenous H_2_O_2_ on the electrophysiological kinetics of N-type Ca^2+^ channels will be addressed in future studies. At last, except voltage-gated Ca^2+^ channels, K^+^ channels also play an important role in the cell excitability of CVP neurons through affecting the repolarization phase of the action potential (111, 112). Although transient outward and inwardly rectifying K^+^ channels are not found in CVP neurons, delayed outward K^+^ channels, including Ca^2+^-dependent K^+^ channels and delayed rectifier K^+^ channels, are functionally detectable in isolated CVP neurons in rats (111). It has been reported that H_2_O_2_ reduces the neuronal excitability through increasing Ca^2+^-dependent K^+^ currents and delayed rectifier K^+^ currents in rat CVP neurons (85). Additionally, ATP-sensitive potassium (K_ATP_) channels are also involved in H_2_O_2_-reduced neuronal activity in CVP neurons isolated from dogs (113). Therefore, future studies are needed to clarify if T2DM alters the protein expression and electrophysiological kinetics of Ca^2+^-dependent K^+^, delayed rectifier K^+^, and K_ATP_ channels.

## Conclusion

**Figure 11:**
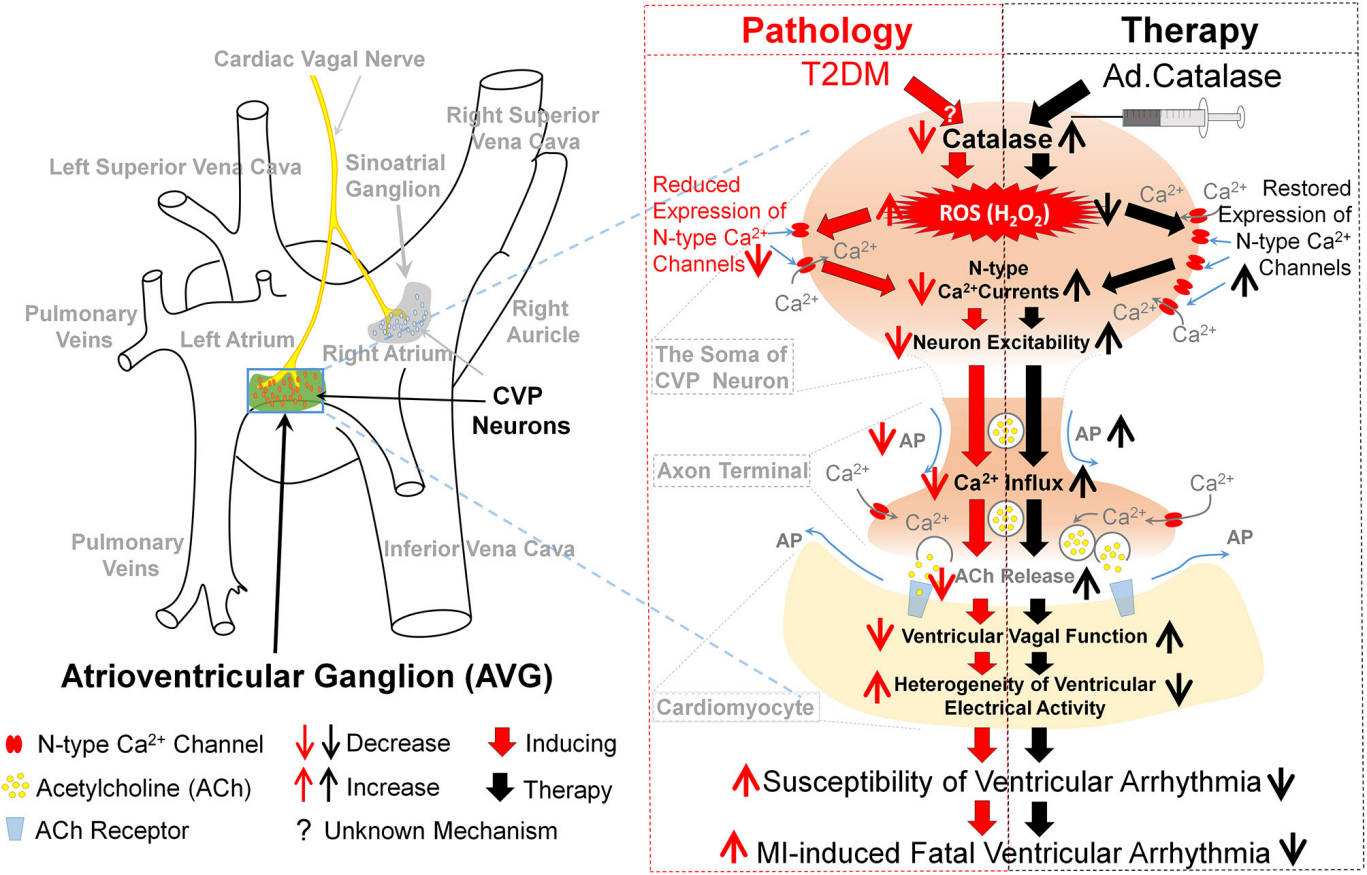
Contribution of endogenous H_2_O_2_ elevation in CVP neurons to ventricular arrhythmogenesis and related therapeutic strategy in T2DM. In the T2DM state, the oxidative stress, as evidenced by endogenous H_2_O_2_ elevation and lowered catalase occurred in CVP neurons. Consequently, endogenous H_2_O_2_ elevation reduced the neuronal excitability through reduction of the N-type Ca^2+^ channel expression and activation. H_2_O_2_ overproduction in CVP neurons further attenuated the cardiac vagal activation and enhanced the susceptibility to ventricular arrhythmogenesis, including the inducibility of ventricular arrhythmias in anesthetized rats and MI-evoked ventricular arrhythmias in conscious rats. Reduction of cytosolic H_2_O_2_ levels in the AVG through *in vivo* Ad.CAT gene transfection markedly increased T2DM-reduced expression and activation of N-type Ca^2+^ channels, intracellular Ca^2+^ levels, and cell excitability of CVP neurons. Ad.CAT gene transfection subsequently improved impaired cardiac vagal function and suppressed ventricular arrhythmogenesis in T2DM. Therefore, targeting H_2_O_2_-N-type Ca^2+^ channel signaling pathway could be a potential therapeutic strategy to improve the withdrawal of cardiac vagal activation and suppress ventricular arrhythmias in T2DM patients with MI.

In summary, our study focused on the H_2_O_2_-N-type Ca^2+^ channel signaling pathway as a novel mechanism for the withdrawal of cardiac vagal activity and ventricular arrhythmogenesis in T2DM. *In vivo* Ad.CAT gene transfection into CVP neurons decreased the cytosolic H_2_O_2_ levels and increased N-type Ca^2+^ currents, intracellular Ca^2+^ levels, and cell excitability of CVP neurons through restoration of the N-type Ca^2+^ channel expression in T2DM rats. Consequently, Ad.CAT gene transfection improved the cardiac vagal function, alleviated the heterogeneity of ventricular electrical activity, and suppressed MI-evoked ventricular arrhythmias in T2DM rats (Figure 11). This study opens a new avenue in therapeutics against the withdrawal of cardiac vagal activity and provides a potential therapeutic strategy for MI-induced lethal ventricular arrhythmias in patients with T2DM.

## Data Availability Statement

The original contributions presented in the study are included in the article/Supplementary Material, further inquiries can be directed to the corresponding author.

## Ethics Statement

The animal study was reviewed and approved by the University of Nebraska Medical Center (UNMC) Institutional Animal Care and Use Committee.

## Author Contributions

DZ and Y-LL conceived and designed the experiments, contributed reagents/materials/analysis tools, and wrote the paper. DZ, HT, WH, BD, MZ, and Y-LL performed the experiments. DZ, HT, WH, and Y-LL analyzed the data. All authors contributed to the article and approved the submitted version.

## Funding

This study was supported by the National Institute of Health's National Heart, Lung, and Blood Institute (R01HL-137832 and R01HL144146 to Y-LL), American Heart Association Career Development Award (851929 to DZ), and Great Plains IDeA-CTR Pilot Grant (to DZ).

## Conflict of Interest

The authors declare that the research was conducted in the absence of any commercial or financial relationships that could be construed as a potential conflict of interest.

## Publisher's Note

All claims expressed in this article are solely those of the authors and do not necessarily represent those of their affiliated organizations, or those of the publisher, the editors and the reviewers. Any product that may be evaluated in this article, or claim that may be made by its manufacturer, is not guaranteed or endorsed by the publisher.
