# Supplementary material for: Hydrogen Peroxide Scavenging Restores N-Type Calcium Channels in Cardiac Vagal Postganglionic Neurons and Mitigates Myocardial Infarction-Evoked Ventricular Arrhythmias in Type 2 Diabetes Mellitus
Source: Front Cardiovasc Med. 2022 Apr 25;9:871852. doi: 10.3389/fcvm.2022.871852 (PMC9082497; doi:10.3389/fcvm.2022.871852)
Supplement: Supplementary file 1 [file Data_Sheet_1.pdf]

SUPPLEMENTAL DATA TO

Hydrogen peroxide scavenging restores N-type calcium channels in cardiac vagal postganglionic neurons and mitigates myocardial infarction-evoked ventricular arrhythmias in type 2 diabetes mellitus

Dongze Zhang,[1†] Huiyin Tu,[1†] Wenfeng Hu,[1†] Bin Duan,[2] Matthew C. Zimmerman,[3] Yu-Long Li[1,3*]

[1]Department of Emergency Medicine, University of Nebraska Medical Center, Omaha, NE 68198, USA;

[2]Mary & Dick Holland Regenerative Medicine Program, Division of Cardiology, Department of Internal Medicine, University of Nebraska Medical Center, Omaha, NE 68198, USA;

[3]Department of Cellular & Integrative Physiology, University of Nebraska Medical Center, Omaha, NE 68198, USA.

†These authors have contributed equally to this work.

*** Correspondence:**

Yu-Long Li, MD, PhD. Department of Emergency Medicine, University of Nebraska Medical Center, 985850 Nebraska Medical Center, Omaha, NE 68198-5850, USA

Tel.: +1-402-559-3016; Fax: +1-402-559-9659; E-mail: yulongli@unmc.edu

Supplemental Figures and Figure legends

Supplemental Figure 1.

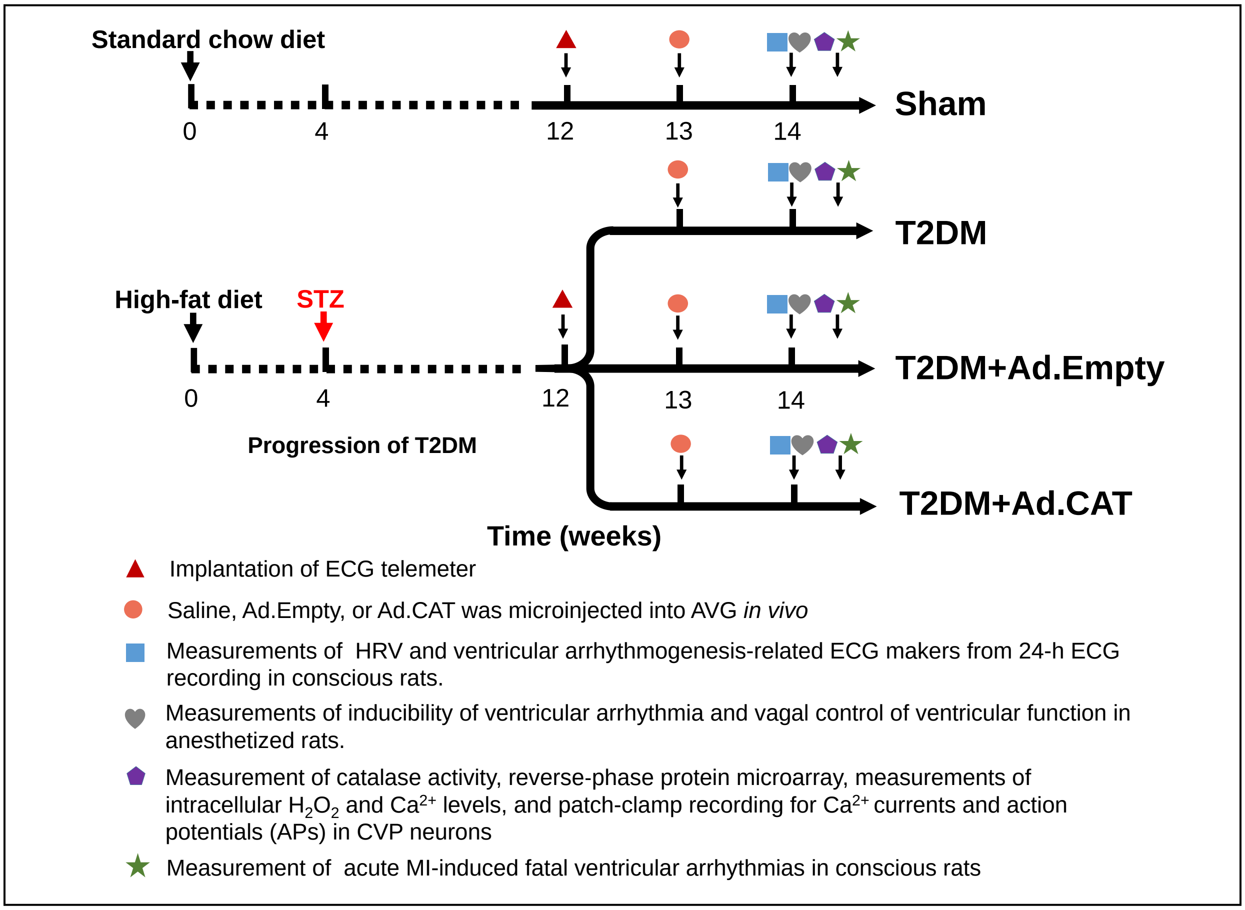

Supplemental Figure 1. Study design, timeline, and interventions. At the beginning of these experiments, rats were randomly assigned to the sham or T2DM group. Sham rats were fed a standard chow diet. T2DM was induced by a combination of high-fat diet followed by low dose streptozotocin (STZ) injection. All experiments were performed at 12-14 weeks of feeding with either standard chow diet or high-fat diet. Implantation of ECG radiotelemetry was performed at the 12th week. T2DM rats were further assigned to three subgroups for different treatments, including T2DM, T2DM+adenoviral vector (Ad.Empty), and T2DM+adenoviral catalase gene (Ad.CAT). Saline, Ad.Empty, or Ad.CAT was microinjected into atrioventricular ganglion (AVG) at the 13th week. Heart rate variability (HRV) and ventricular arrhythmogenesis-related ECG makers were evaluated from 24-hour radiotelemetry ECG recording in conscious rats at 1 week after gene transfection in all groups. Terminal experiments (including measurements of the inducibility of ventricular arrhythmia and vagal control of ventricular function in anesthetized rats, myocardial infarction (MI)-induced fatal ventricular arrhythmia in conscious rats, hemodynamics and morphology, measurement of catalase activity, reverse-phase protein microarray, intracellular H_2O_2 and Ca^{2+} levels, and whole-cell patch-clamp recording in CVP neurons) were performed at the 14th week.

Supplemental Figure 2

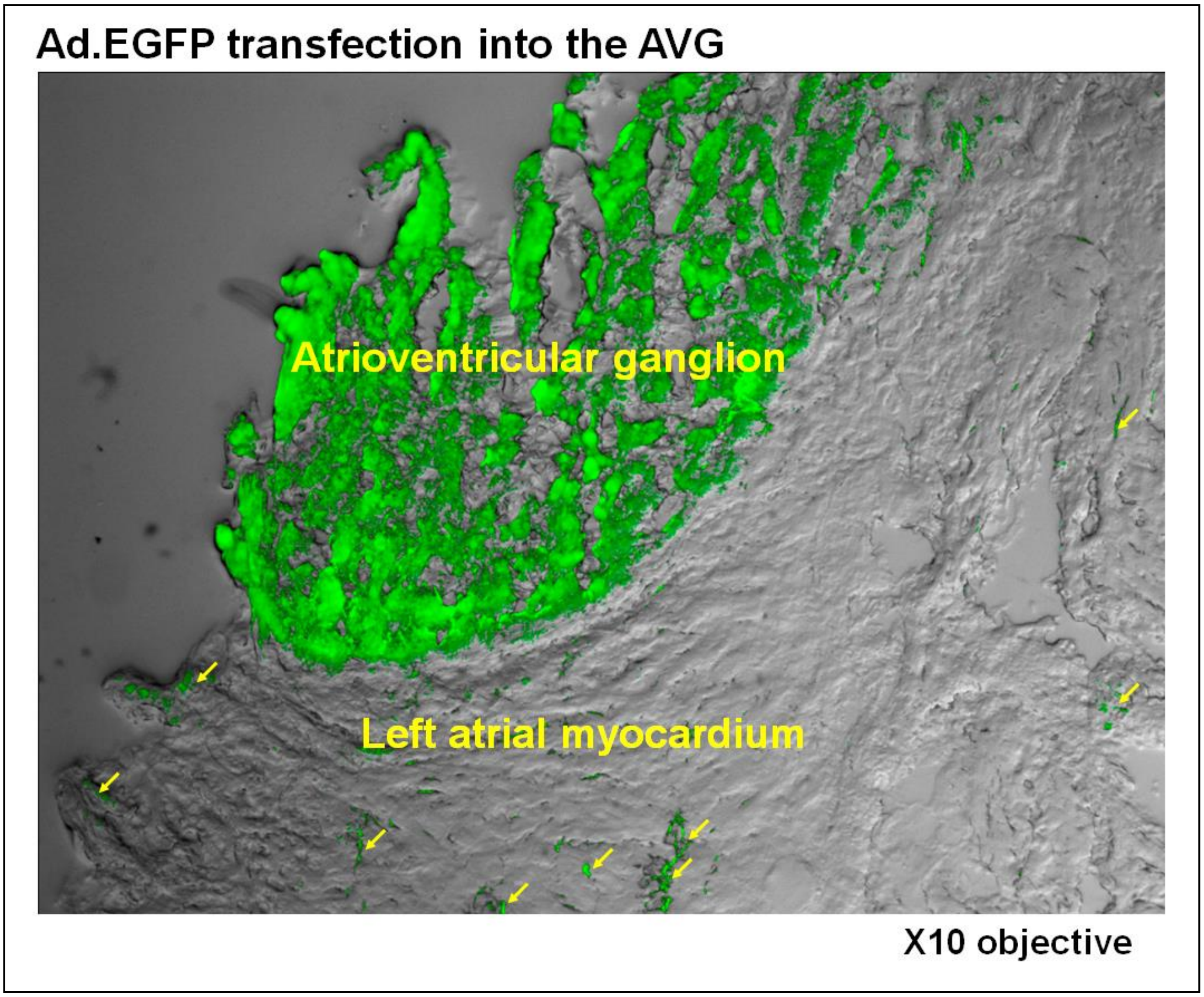

Supplemental Figure 2. Adenoviral mediated transfection of enhanced green fluorescent protein (Ad.EGFP, green color) to the AVG in a T2DM rat. Background is bright field monochrome image of AVG area with the left atrial myocardium. Yellow arrows indicate nerve fibers.

Supplemental Figure 3

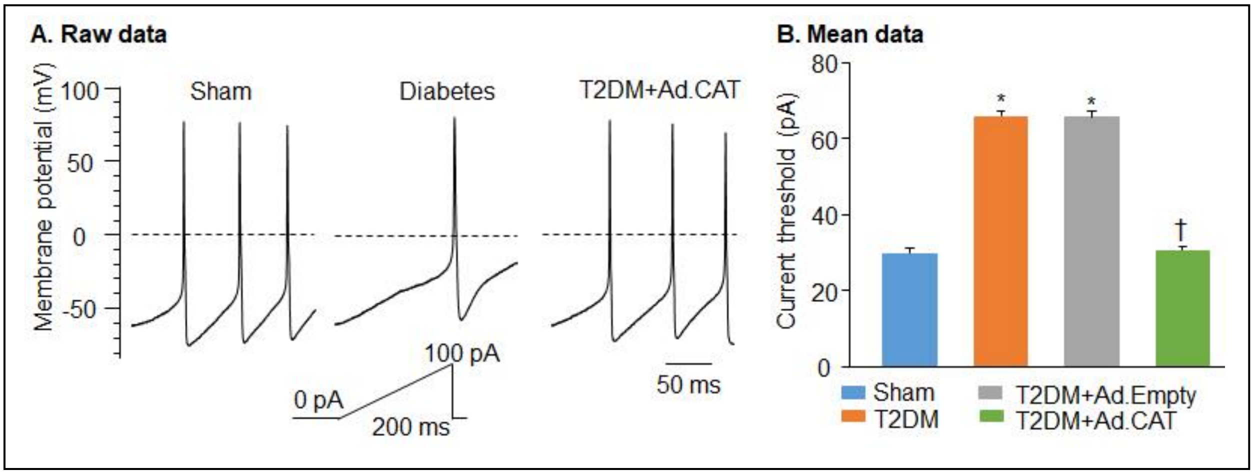

Supplemental Figure 3. Original recording (A) and mean data (B) of current threshold-inducing action potential in CVP neurons from all groups. Action potential was elicited by a ramp current injection of 0-100 pA in the current-clamp mode. The current threshold-inducing action potentials was measured at the beginning of the first action potential. N=8 neurons from 6 rats per group; data means ± SEM. Statistical significance was determined by one-way ANOVA with post-hoc Bonferroni test. *P<0.05 vs. sham; †P<0.05 vs. T2DM.

Supplemental Table 1. Metabolic characteristics of sham and T2DM rats

	Sham (n=32)	T2DM (n=96)
Body Weight (g)	412.1±7.4	357.4±5.6*
Fasting Blood Glucose (mg/dl)	92.5±6.3	458.9±7.3*

Data are means ± SEM. Statistical significance was determined by student's unpaired t-test. *P < 0.05 vs. Sham.

Supplemental Table 2. Electrophysiological properties in CVP neurons from all groups of rats

	RMP (mV)	R_i (GΩ)	Cm (pF)
Sham	-61.4 ± 1.5	0.82 ± 0.05	34.9 ± 1.8
T2DM	-60.9 ± 1.4	0.81 ± 0.06	35.3 ± 2.3
T2DM+Ad.Empty	-61.2 ± 1.8	0.83 ± 0.06	34.6 ± 2.4
T2DM+Ad.CAT	-61.6 ± 1.9	0.81 ± 0.06	35.5 ± 2.1

Data are means ± SEM. RMP, resting membrane potential; Ri, input resistance; Cm, membrane capacitance. N = 8 neurons from 6 rats per group.

Supplemental Table 3. Hemodynamic characteristics in all groups of rats

	Sham (n=8)	T2DM (n=8)	T2DM+ Ad.Empty (n=8)	T2DM+ Ad.CAT (n=8)
MBP (mmHg)	105.5± 3.3	103.1±3.8	105.1±2.8	103.3±3.6
HR (bpm)	354.9±9.3	353.1±11.8	356.6±10.7	352.8±11.1
LVSP (mmHg)	122.4±2.5	120.9±3.4	119.5±3.1	117.4±3.3
LVEDP (mmHg)	1.8±0.3	1.8±0.1	1.8±0.2	1.9±0.2
LV dP/dt_{max} (mmHg/s)	6107.6±175.1	5882.9±185.2	5877.1±151.2	5976.9±177.1

Data are means ± SEM. MBP: mean blood pressure; HR: heart rate; LVSP: left ventricular end-systolic pressure; LVEDP: left ventricular end-diastolic pressure; LV dp/dt_{max}: the maximum rate of increase of left ventricular pressure.
